# Acetyl-CoA synthetase 2 contributes to a better prognosis for liver cancer by switching acetate-glucose metabolism

**DOI:** 10.1038/s12276-024-01185-3

**Published:** 2024-03-25

**Authors:** Kyung Hee Jung, Sujin Lee, Han Sun Kim, Jin-Mo Kim, Yun Ji Lee, Min Seok Park, Myeong-Seong Seo, Misu Lee, Mijin Yun, Sunghyouk Park, Soon-Sun Hong

**Affiliations:** 1 Department of Biomedical Sciences, College of Medicine, and Program in Biomedical Science & Engineering, Inha University, 3-ga, Sinheung-dong, Jung-gu, Incheon, 22332 Korea; 2 Department of Manufacturing Pharmacy, Natural Products Research Institute, College of Pharmacy, Seoul National University, Seoul, 08826 Korea; 3 Division of Life Science, College of Life Science and Bioengineering, Incheon National University, Incheon, 21999 Korea; 4 Department of Nuclear Medicine, Severance Hospital, Yonsei University College of Medicine, 134 Shinchon-dong, Seodaemun-gu, Seoul, 03722 Korea

**Keywords:** Cancer metabolism, Targeted therapies

## Abstract

Acetyl-CoA synthetase 2 (ACSS2)-dependent acetate usage has generally been associated with tumorigenesis and increased malignancy in cancers under nutrient-depleted conditions. However, the nutrient usage and metabolic characteristics of the liver differ from those of other organs; therefore, the mechanism of ACSS2-mediated acetate metabolism may also differ in liver cancer. To elucidate the underlying mechanisms of ACSS2 in liver cancer and acetate metabolism, the relationships between patient acetate uptake and metabolic characteristics and between ACSS2 and tumor malignancies were comprehensively studied in vitro*,* in vivo and in humans. Clinically, we initially found that ACSS2 expression was decreased in liver cancer patients. Moreover, PET-CT imaging confirmed that lower-grade cancer cells take up more ^11^C-acetate but less ^18^F-fluorodeoxyglucose (^18^F-FDG); however, this trend was reversed in higher-grade cancer. Among liver cancer cells, those with high ACSS2 expression avidly absorbed acetate even in a glucose-sufficient environment, whereas those with low ACSS2 expression did not, thereby showing correlations with their respective ACSS2 expression. Metabolomic isotope tracing in vitro and in vivo revealed greater acetate incorporation, greater lipid anabolic metabolism, and less malignancy in high-ACSS2 tumors. Notably, ACSS2 downregulation in liver cancer cells was associated with increased tumor occurrence in vivo. In human patient cohorts, patients in the low-ACSS2 subgroup exhibited reduced anabolism, increased glycolysis/hypoxia, and poorer prognosis. We demonstrated that acetate uptake by ACSS2 in liver cancer is independent of glucose depletion and contributes to lipid anabolic metabolism and reduced malignancy, thereby leading to a better prognosis for liver cancer patients.

## Acetyl-CoA Synthetase 2: A New Hope for Liver Cancer Prognosis

Liver cancer patients with high amounts of Acetyl-CoA synthetase 2 (ACSS2), an enzyme that helps break down acetate, tend to have a better outlook than those with low amounts, according to a study by Kyung Hee Jung and team. The researchers discovered that high ACSS2 levels are linked with anabolic characteristics, reduced glycolysis, and lower hypoxia, which all point to less severe cancer. The study involved lab experiments, animal testing, and analysis of human patient data. The team also found a group of patients with very low ACSS2 levels who had especially bad outlooks. The results suggest that ACSS2 could be a potential indicator for liver cancer outlook and could assist in making treatment choices.

This summary was initially drafted using artificial intelligence, then revised and fact-checked by the author.

## Introduction

Glucose is considered the preferred nutrient for cancer cells according to Otto Warburg’s proposal of aerobic glycolytic properties for all growing cancer cells^1^. However, other nutrients, such as glutamine, lactate, and acetate, can also be utilized by tumors under certain environmental conditions^2–4^. Acetate usage depends on acetyl-CoA synthetase 2 (ACSS2); specifically, the use of acetate in cancers seems to occur mostly under nutrient-depleted or hypoxic conditions^4–6^. Another aspect of acetate uptake is its relationship with tumor malignancy. A higher level of ACSS2 is associated with a shorter survival time and metastasis in patients with brain, breast, or prostate cancers^7,8^. Bioinformatic analyses have suggested links between increased acetate utilization and higher malignancy under hypoxic conditions^9,10^.

Although the above studies suggest that hypoxia/nutrient depletion is positively correlated with cancer and that hypoxia/nutrient depletion and higher acetate uptake are positively correlated with cancer malignancy, there is contrasting evidence. It has been established that a high glucose uptake, which might limit acetate uptake, can be induced under hypoxic conditions via hypoxia-inducible factors associated with increased cancer malignancy^11,12^. Acetate metabolism through ACSS2 seems to be important in leukemogenesis, although blood cancer cells are exposed to nutrient- and oxygen-rich environments^13^. Positron emission tomography (PET) imaging has shown that well-differentiated liver cancer is better detected with ^11^C-acetate, whereas poorly differentiated liver cancer is better detected with ^18^F-fluorodeoxyglucose (^18^F-FDG)^14^. Therefore, there is a lack of clarity regarding the relationships between acetate uptake, nutrient availability and cancer malignancy; moreover, these relationships may differ based on cancer type and stage.

The nutrient usage and metabolic characteristics of the liver may differ from those of other organs, as exemplified by its synthesis of hepatic glucose and cholesterol relative to their consumption in other organs^15^. Given the unique metabolic nature of the liver and the controversies stated above, the relationship between acetate uptake, metabolic characteristics, and liver cancer malignancy cannot be extrapolated from other tumors and has yet to be fully elucidated. Therefore, in this study, we clarified the correlation between acetate metabolism and liver cancer malignancy via positron emission tomography (PET)-CT and transcriptomics and through a clinical analysis of liver cancer patients. Furthermore, we verified the acetate metabolism via ACSS2 in vitro and in vivo by performing isotopic tracing.

## Materials and methods

### Chemicals and reagents

The stable isotope-labeled sodium acetate (CLM-440-1, 1,2-^13^C_2_, 99%) and D-glucose (CLM-1396-5, U-^13^C_6_, 99%) were purchased from Cambridge Isotope Laboratories (Andover, MA, USA). The inhibitor of ACSS2, 1-(2,3-di(thiophen-2-yl)quinoxalin-6-yl)-3-(2-methoxyethyl)urea (7032008), was purchased from ChemBridge (San Diego, CA, USA). The carnitine palmitoyltransferase I (CPT1) inhibitor (etomoxir, 11969) was purchased from Cayman Chemical (Ann Arbor, MI, USA). The ACSS2 antibody (PA5-52059) was obtained from Thermo Fisher Scientific (Waltham, MA, USA), and the β-actin antibody (sc-47778) was obtained from Santa Cruz Biotechnology (Santa Cruz, CA, USA). N-nitrosodiethylamine (DEN) was purchased from Sigma‒Aldrich (St. Louis, MO, USA).

### Cell culture, isotope labeling, and nutrient uptake

Cells were cultured in accordance with the following standard procedures. The cell lines were regularly tested for mycoplasma contamination using the “Myco-Read Mycoplasma Detection Kit” (Biomax, Seoul, Korea). U-^13^C_6_-D-glucose (5 mM) (with unlabeled 0.2 mM acetate) and/or 0.2 mM 1,2-^13^C_2_-sodium acetate (with unlabeled 5 mM glucose) were added as necessary. For isotope uptake, the cells were incubated for 16 h with the aforementioned isotopes in the media. Uptake was evaluated based on the peak intensities of the remaining 1,2-^13^C_2_-acetate or U-^13^C_6_-glucose in the media using 1D and heteronuclear nuclear magnetic resonance (NMR) spectroscopy.

### Metabolite extraction

The cells (2 × 10^7^) were harvested using trypsin-ethylenediaminetetraacetic acid (Gibco, Grand Island, NY, USA) and centrifuged. The harvested cells were resuspended in 900 μL of methanol-chloroform solution (2:1, v/v). After three cycles of liquid nitrogen-thaw shaking (20 min), 300 μL of chloroform and 300 μL of distilled water were added to the cell lysates. The samples were then centrifuged at 21,000 × *g* for 10 min at 4 °C. The lipid layer and water layer were dried separately using a vacuum centrifuge (Vision, Seoul, Korea). For the NMR measurements, the pellet was resuspended in 500 μL of a buffer comprising 2 mM Na_2_HPO_4_ and 5 mM NaH_2_PO_4_ in D_2_O with 0.025% trimethylsilylpropionic acid sodium salt-d4 (TSP). For the liquid chromatography–mass spectrometry (LC–MS) measurements, the pellet was reconstituted with 40 μL of acetonitrile:distilled water (1:1, v/v), and another 2 μL was introduced after centrifugation at 21,000 × *g* for 10 min at 4 °C. The mouse tissues were cut to a size of 100 mg and extracted in the same way as the cell extraction method after homogenization with a disposable homogenizer (BioMasher, Nipi, Tokyo, Japan).

### Nuclear magnetic resonance (NMR)

The data were obtained using an 800-MHz Bruker Avance III HD spectrometer equipped with a 5-mm CPTCI Cryoprobe (Bruker BioSpin, Germany). 2D heteronuclear single quantum coherence (HSQC) spectra were obtained using the Bruker pulse sequence hsqcetgpsisp2.2. The dataset comprised 1024 × 512 complex points. For each experiment, five scans were performed per T1 increment for 95 min. For the glucose and acetate uptake experiments, each medium was collected and centrifuged at 21,000 × *g* for 10 min. The supernatants were mixed with a 10% buffer composed of 2 mM Na_2_HPO_4_ and 5 mM NaH_2_PO_4_ in D_2_O supplemented with 0.025% TSP. The NMR spectra were obtained with an 800-MHz NMR machine using a 1D HSQC (TD 16384) experiment.

### Liquid chromatography–mass spectrometry (LC–MS) measurement of the isotopomer distribution

The samples were analyzed using an Acquity ultraperformance LC system (Waters Corp., Milford, MA) with a SeQuant® ZIC®-pHILIC polymeric bead polyetheretherketone column (150 × 2.1 mm, 5 μm; Merck, Darmstadt, Germany). Ammonium carbonate (10 mM, pH = 9) in distilled water (A) and acetonitrile (B) were used as the mobile phases, and the flow rate was 0.15 mL/min. The gradients used were as follows: 20% A at 0 min, 20% A at 2 min, 80% A at 19 min, 80% A at 30 min, 20% A at 30.5 min, and 20% A at 35 min. Hydrophobic sample separation was performed on a Kinetex C18 column (100 × 4.6 mm, 2.6 μm; Phenomenex, CA, USA). Here, 60:40 (v/v) acetonitrile:distilled water with 10 mM ammonium acetate (A) and 90:10 (v/v) isopropanol:acetonitrile with 10 mM ammonium acetate (B) were used as the mobile phases, with a flow rate of 0.4 mL/min. The gradients used were as follows: 85% A at 0 min, 70% A at 3.25 min, 52% A at 4 min, 18% A at 16.75 min, 1% A at 17.5 min, 1% A at 18.25 min, 85% A at 18.40 min, and 85% A at 23 min. The MS data were obtained with a Q Exactive^TM^ Focus Hybrid Quadrupole-Orbitrap^TM^ mass spectrometer (Thermo Scientific, Waltham, MA, USA).

### NMR and LC‒MS measurements of the isotopomer distribution

For the NMR measurements, if applicable, the number of nonuniform sampling points was determined to be 128 complex points (25% of the sampling density of 512 points) using the nuslist, as described previously^16^. The isotopomer distribution was obtained from the LC‒MS peak intensities after natural abundance correction, and the relative abundance was expressed as a percentage relative to the sum of all isotopomer intensities, including M + 0.

### Fatty acid oxidation

To evaluate fatty acid oxidation, 10^6^ HepG2 or Hep3B cells were seeded in six-well plates with D-glucose-free DMEM supplemented with 10% FBS, 1% penicillin/streptomycin, and 5.5 mM ^13^C_6_-glucose. The fatty acid ω-carbons were labeled with the ^13^C isotope for 24 h. On the next day, the ^13^C-glucose-containing media were removed, and the plates were washed with DPBS. For the 0 h control group, the hydrophobic metabolites were extracted using a two-layer methanol–chloroform extraction method. For the 24 h test groups, the cells were further incubated for another 24 h in D-glucose-free DMEM supplemented with 10% FBS, 1% penicillin/streptomycin, and 5.5 mM ^12^C-glucose. The hydrophobic metabolites of the 24 h test group were extracted using the same extraction method. The decrease in the labeled fatty acid ω-carbon content was measured by 800-MHz NMR using an HSQC experiment. Fatty acid oxidation was evaluated as the relative peak intensity decrease in ω-carbon content at the 24 h time point relative to that at 0 h.

### Fluorescent signal quantification

Equal numbers of cells were plated on confocal dishes (200350, SPL Life Sciences, Gyeonggi-do, Korea). The cells were stained with Hoechst 33342, MitoTracker^TM^ Red CMXRos, and ^Bodipy^ FL C_12_ (Thermo Fisher Scientific, Waltham, MA, USA) for 15 min. The stained cells were washed with Dulbecco’s phosphate-buffered saline (DPBS) and fixed with 4% paraformaldehyde solution for 30 min at room temperature. The fluorescent signals were measured using a TCS8 confocal microscope (Leica Microsystems, Wetzlar, Germany).

### Wound healing and clonogenic assays

A wound healing assay was also conducted to assess cell migration. The lines were generated with a sterile 1-mL pipette tip. At 0, 24, 48, and 72 h, images were taken using an AmScope microscope digital camera MD800E (Irvine, CA, USA). The wound healing area was measured using ImageJ software. HepG2 or Hep3B cells (800 per well) were seeded into a six-well plate and cultured in Dulbecco’s modified Eagle’s medium (DMEM) supplemented with 10% fetal bovine serum (FBS) for 2 weeks. Then, the cells were fixed and stained with a crystal violet (0.5% w/v) solution in 20% methanol. The colonies were then counted.

### Small interfering RNA (siRNA)-mediated knockdown

Predesigned small interfering RNAs (siRNAs) targeting *ACSS2* (55902-1) were purchased from Bioneer (Daejeon, Republic of Korea). The cells were transfected with 100 nM siRNA using Lipofectamine RNAiMAX for 48 h (Thermo Fisher Scientific) according to the manufacturer’s protocol. The media were replaced with fresh media, and colorimetric cell viability assays were conducted after 24 h using a D-Plus^TM^ cell-counting kit (CCK-3000; Dongin Biotech, Seoul, Korea). The cellular viability was measured by measuring the absorbance at 450 nm using a microplate reader (Molecular Devices, San Jose, CA, USA).

### Short hairpin RNA (shRNA) experiment

HEK293T cells were plated in a dish and transfected with a human ACSS2 short hairpin RNA (shRNA) lentiviral vector or control vector (Sigma‒Aldrich; TRCN0000045564, SHC016). The lentiviral particles were harvested after incubating for 48 h. The HepG2 cells were seeded into a dish at approximately 40% confluence. The cells were exposed to the prepared lentiviral particles along with 5 μg/mL polybrene for 24 h, after which the medium was replaced with fresh complete medium. After incubating for 48 h, the stable clones were selected using 10 μg/mL puromycin. Single-cell selection was performed to ensure homogeneity. The knockdown of ACSS2 was confirmed by western blotting.

### Generation of ACSS2-overexpressing cells

HEK293T cells were plated in a dish and transfected with a human pLenti-GIII-CMV-ACSS2 lentiviral vector or control vector (abm, Richmond, BC, USA; LV707079). The lentiviral particles were harvested after incubating for 48 h. The Hep3B cells were seeded into a dish at approximately 50% confluence. The cells were exposed to the prepared lentiviral particles along with 5 μg/mL polybrene for 24 h, after which the medium was replaced with fresh complete medium. After incubating for 48 h, the stable clones were selected using 5 μg/mL puromycin. Single-cell selection was performed to ensure homogeneity. ACSS2 overexpression was confirmed by western blotting.

### Western blotting

The cells were lysed with radioimmunoprecipitation assay (RIPA) buffer (P3200-010; Biosesang, Gyeonggi-do, Korea) containing protease and phosphatase inhibitor cocktails (GenDepot, Barker, TX, USA). The proteins were resolved by sodium dodecyl sulfate‒polyacrylamide gel electrophoresis (SDS‒PAGE) and transferred onto nitrocellulose membranes. The blots were immunostained with appropriate primary antibodies, followed by incubation with secondary antibodies conjugated to horseradish peroxidase. Antibody binding was detected with an enhanced chemiluminescence reagent (Bio-Rad. Hercules, CA, USA). Primary antibodies against the following molecules were used: ACSS2 (Thermo Fisher Scientific) and β-actin (Santa Cruz Biotechnology). The secondary antibodies were purchased from Santa Cruz Biotechnology.

### qRT‒PCR

RNA was incubated in the presence of poly(A) polymerase (PAP; Takara, Kusatsu, Japan), MnCl_2_, and ATP for 1 h at 37 °C. Then, reverse transcription was performed using an oligodT primer harboring a consensus sequence on total RNA (5 µg) with SuperScript II RT (Invitrogen, Carlsbad, CA, USA). Next, the cDNA was amplified by RT‒PCR; SYBR Green qRT‒PCR was performed on a Step ONE Plus (Applied Biosystems, Forster City, CA, USA). In each run, 1 µL of cDNA was used as a template for amplification per reaction. The sample was added to 19 µL of reaction mixture containing 7 µL of H_2_O, 10 µL of QuantiTect^®^ SYBR^®^ Green PCR Master Mix (Qiagen, Hilden, Germany) and 1 µL of forward and reverse primers (supplementary Table 1). Real-time qRT‒PCR amplification of the genes was carried out for 35 cycles of 95 °C for 15 sec and 59 °C for 1 min after 95 °C for 15 min. Three independent experiments were performed.

### Orthotopic tumor implantation in mice

BALB/c nude mice (8–9 weeks old) were anesthetized with isoflurane, and the skin was sterilized with iodophor three times before surgery. Small-volume suspensions containing the cells (3 × 10^6^) and Matrigel were injected into the subcapsular region of the liver parenchyma in the median lobe. Immediately before injection, the cell suspension was mixed at a 1:1 (vol/vol) ratio with the Matrigel in a small tube to increase the viscosity of the injected cell suspension. Small syringes (0.3 mL) with 31-gauge needles were used to inject the cell/Matrigel suspension. To avoid leakage of tumor cells from the injection site, the injection volume was limited to 30 μL. A steady and slow injection was performed to prevent leakage of the injected cell suspension. After the injection, the site was gently pressed with cotton balls to reduce bleeding and leakage of the cell suspension. After two months, the mice were sacrificed, and the tumors were isolated. Segments of visible tumors were carefully excised and immediately frozen in liquid nitrogen until analysis.

### Glucose and acetate metabolism in orthotopic liver cancer models

Following the Institutional Animal Ethics Committee’s permission at Inha University (INHA-181120-600-1), four-week-old male BALB/c nude mice were purchased from Orient Bio (Seoul, Korea). The human liver cancer orthotopic nude mouse models with Hep3B, HepG2, and shACSS2-HepG2 cells were established as previously described^17^. The visible tumor tissues were carefully excised and analyzed. For the experiments in which isotopically labeled acetate and glucose were administered to the animals, the mice were fasted for at least 12 h and orally administered U-^13^C_6_ glucose (5 g/kg, 300 μL) or 1,2-^13^C_2_ acetate (3 g/kg, 300 μL) for the orthotopic xenograft models. The animals were euthanized 30 and 120 min after the administration of glucose and acetate, respectively. Tissues were collected, rapidly frozen to quench metabolism and stored at −80 °C for further analysis. With a single-bolus injection, glucose absorption was faster than that with acetate, and thus, its appearance and disappearance were faster than those with acetate. We tested multiple time points and selected the optimal time point for each tracer.

### N-nitrosodiethylamine (DEN)-induced liver cancer model

For the rat liver cancer model, male Sprague‒Dawley rats weighing 150–180 g were administered DEN in their drinking water (100 mg/L) and then sacrificed at the indicated times (13 and 17 weeks posttreatment). All the rats developed tumors, and the tumor tissues were excised to compare their ACSS2 expression with that of normal tissue.

### Immunohistochemistry (IHC) of human liver cancer tissues

Human tissue microarrays for patients with normal (*n* = 22), cirrhosis (*n* = 28), liver cancer (*n* = 69), and liver metastasis (*n* = 15) (US Biomax, Inc., Rockville, MD, USA) were used to examine ACSS2. Primary tumors and metastases were classified according to TNM staging, which is used to determine the prognostic value of cancer. The expression of ACSS2 was statistically analyzed according to the pathology grade of the samples. In brief, two independent observers, including one pathologist who was blinded to the clinical data, examined and scored all the tissue specimens. The staining intensity and the proportion of positive cells were measured, and staining scores were assigned as follows: [IHC score 1], weak staining in <50% or moderate staining in <20% of stromal cells; [IHC score 2], weak staining in ≧50%, moderate staining in 20–50% or strong staining in <20%; and [IHC score 3], moderate staining in ≧50% or strong staining in ≧20%. In cases of discrepancies, a final score was established by reassessment by both pathologists using a double-headed microscope. For this experiment, the tissue array slides were deparaffinized with xylene and dehydrated with ethanol. Antigen retrieval was performed by microwave heating in a citrate solution for 20 min. A blocking solution was used to prevent nonspecific antibody binding. The tissue sections were incubated with a polyclonal anti-ACSS2 antibody (1:50 dilution) overnight at 4 °C. A horseradish peroxidase detection system (HRP streptavidin label and polyvalent biotinylated link) and diaminobenzidine substrate kit were used as the detection reagents (Vector Laboratories, Burlingame, CA, USA). After counterstaining with hematoxylin, the sections were dehydrated and mounted. All of the ACSS2-stained slides were scanned using the ScanScope System (Leica, Wetzlar, Germany).

### Positron emission tomography–computed tomography (PET-CT) imaging

Approximately 5.5 MBq of ^18^F-FDG was administered intravenously per kilogram of body weight. Then, 60 min after the injection, PET-CT scanning was performed from the skull base to the mid-thigh in 3D mode at 2 min per bed position using a dedicated PET/CT scanner (Discovery 600; General Electric Medical Systems, Milwaukee, WI, USA). Low-dose CT was performed using the following parameters: a scout view at 10 mA and 120 kVp, followed by a spiral CT scan with a 0.8-s rotation time, 60 mA, 120 kVp, a 3.75-mm section thickness, 1.25-mm collimation, and 27.5-mm table feed per rotation with the arms raised. The CT images were reconstructed onto 512 × 512 matrices and converted into 511 keV-equivalent attenuation factors for attenuation correction. The PET images were reconstructed into 128 × 128 matrices using ordered subset expectation maximization and were corrected for random and scatter coincidences. In addition, a separate PET‒CT scan was obtained using the same scanner 20 min after an intravenous injection of 370–555 MBq of ^11^C-acetate at 2 min per bed position. The same attenuation correction and image reconstruction were performed for the ^11^C-acetate images. Comparisons of higher- and lower-grade cancers were made for patients who underwent tumor resection for surgical treatment, and their tissues were evaluated via histological examination.

### Gene set enrichment analysis (GSEA)

For the gene set enrichment analysis (GSEA) between the HepG2 and Hep3B cell lines, the signal values of the GSE21955 dataset were downloaded, and an offset of 34.1226 was added to the entire dataset of values to make them all positive. The data were log_2_ transformed, and the probes were collapsed into gene symbols. When GSEA was performed, only the gene sets related to metabolism were used. These gene sets were obtained by downloading all pathways from Reactome (www.reactome.org) and extracting only those pathways under the “Metabolism” hierarchy. For the GSEA between the high-ACSS2 and low-ACSS2 patient groups, the same metabolic gene sets were used. For the GSEA shown in Supplementary Fig. 6, the same GSEA method was used as that for the HepG2 and Hep3B cell lines shown in Supplementary Fig. 2, except that the gene set used was “h.all.v7.1.symbols.gmt” in the Molecular Signatures Database.

### Survival analysis

**Fig. 1:**
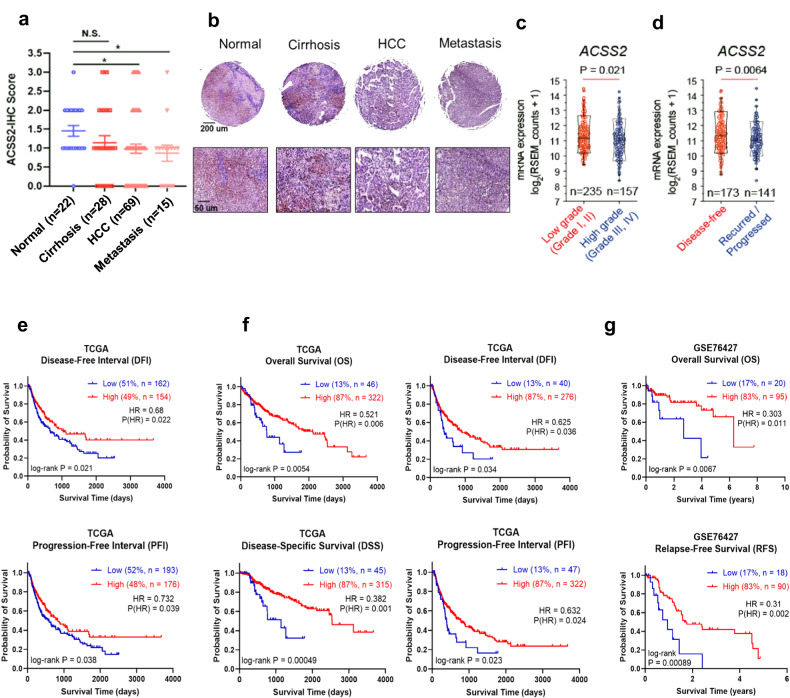
Clinical manifestations of high- and low-acetyl-CoA synthetase 2 (ACSS2) in human liver cancer patients. **a** Immunohistochemistry analysis of ACSS2 in a human liver tissue microarray. Staining intensity was graded as 0 (none), 1+ (weak) (<30%), 2+ (moderate) (+30–60%), or 3+ (>60%). **b** Representative images of immunohistochemical staining of liver tissues. **c**, **d** ACSS2 mRNA expression levels in patients in the Cancer Genome Atlas (TCGA) liver hepatocellular carcinoma (LIHC) cohort with low-grade (I and II) vs. high-grade (III and IV) tumors or disease-free vs. recurrent/progressive patients. **e**–**g** K‒M analysis of prognostic variables in patients from the TCGA-LIHC cohort. **e** Disease-free interval (DFI) and progression-free interval (PFI) curves based on the 2352 “RNA-Seq by Expectation-Maximization” (RSEM) cutoff. **f** OS, disease-specific survival (DSS), DFI, and PFI for the low-ACSS2 (13VLA group) and high-ACSS2 (the remaining) groups. HR: hazard ratio. **g** OS and recurrence-free survival (RFS) curves for the bottom 17% and top 83% of the ACSS2 patients in the GSE76427 dataset.

For survival analysis, the ‘survival’ and ‘survminer’ R packages were used. The log-rank test and Cox proportional hazards regression were used for the comparison of curves and hazard differences, respectively. For stratification of the patients, in the DFI analysis shown in Fig. 1e, the surv_cutpoint function with the ‘minprop’ parameter set to 0.47 was used. This yielded 2352 RSEMs, which was also used as the cutoff value for the PFI analysis, as shown in Fig. 1e. In the OS analysis shown in Fig. 1f, the surv_cutpoint function with the ‘minprop’ parameter set to 0.1 was used. This gave the threshold of lower 13th percentiles, which was also used as the cutoff for the DFI, DSS, and PFI analyses, as shown in Fig. 1f. In the OS analysis shown in Fig. 1g, the surv_cutpoint function with the ‘minprop’ parameter set to 0.1 was used. This gave the threshold of the lower 17th percentile, which was also used as the cutoff in the RFS analysis shown in Fig. 1g.

### Clinical data sources

The messenger RNA (mRNA) expression levels (normalized counts of “RNA-Seq by Expectation-Maximization” [RSEM]) were obtained from The Cancer Genome Atlas (TCGA) Pan-Cancer Atlas (https://gdc.cancer.gov/about-data/publications/pancanatlas). Survival information was obtained from the TCGA-Clinical Data Resource. The tumor grade information was obtained from the TCGA Genomic Data Commons (GDC) portal (http://portal.gdc.cancer.gov). Disease-free status information was obtained from Bioportal (www.cbioportal.org). The hypoxia score was also downloaded from Bioportal (www.cbioportal.org).

### Methylation analysis

The beta values of the TCGA liver hepatocellular carcinoma (LIHC) cohort were obtained from the TCGA Pan-Cancer Atlas (https://gdc.cancer.gov/about-data/publications/pancanatlas). For each probe, the difference in the median beta values between the high-ACSS2 subgroup and the low-ACSS2 subgroup was calculated. The *P* value was calculated using the Wilcoxon rank-sum test, and the false discovery rate (FDR) was determined using the Benjamini–Hochberg method. Then, for each probe, the values of −log_10_(FDR) were obtained and plotted by multiplying by the difference in the medians of the beta values. Among the multiple probes for the *ACSS2* or fatty acid synthase (*FASN*) genes, the probe that showed the strongest negative correlation (by Spearman’s rank correlation) with gene expression was selected and marked as diamond (*ACSS2* gene) or square (*FASN* gene) in Supplementary Fig. 12. For *ACSS2*, the probe was “cg09801828,” and for the *FASN* gene, the probe was “cg25068915.”

### Statistical analysis

The Wilcoxon-rank sum test, Student’s *t* test, or Welch’s *t* test was used to compare two continuous variables. Student’s *t* test or Welch’s *t* test was used depending on the *p* value of the *F* test (threshold of 0.05). For survival analysis, the log-rank test and Cox proportional hazards regression were used (details are provided in the ‘Survival analysis’ section). For human HCC tissue scores, we used the nonparametric Jonckheere-Terpstra test to test the population median order of scores in the normal, cirrhotic, HCC and metastatic groups. The statistical analysis was performed using R Software Version 3.6.2. The error bars in the bar graphs represent the standard deviation.

## Results

### Clinical manifestations of high- and low-ACSS2 liver cancer patients

To determine the expression of ACSS2 during the course of human liver tumorigenesis, we initially compared the ACSS2 levels in tissues from normal, cirrhotic, hepatocellular carcinoma, and metastatic liver cancer. The staining intensity and the proportion of positive cells were measured, and staining scores were assigned as described in the “Methods” section. Immunohistochemical staining revealed that ACSS2 expression was significantly greater in normal tissues than in hepatocellular carcinoma and metastatic liver cancer (Fig. 1a, b), indicating that the ACSS2 expression is lower in malignant tumors than in normal tissues. Consistent results were also obtained in a DEN-induced rat liver cancer model, in which ACSS2 expression decreased as the tumor developed (Supplementary Fig. 1a, b).

The ACSS2-malignancy relationship was further tested in a larger cohort of liver cancer patients derived from TCGA. ACSS2 expression was greater in lower-grade tumors (grades I and II) than in higher-grade tumors (grades III and IV) (Fig. 1c). In addition, patients with cancer recurrence and/or progression had lower ACSS2 levels than did those who achieved disease-free status (Fig. 1d). Furthermore, the progression-free interval (PFI) and disease-free interval (DFI) were significantly longer in the high-ACSS2 subgroup than in the low-ACSS2 subgroup (Fig. 1e) when examining similarly sized high- and low-ACSS2 groups with the RSEM cutoff value of 2352. An initial patient survival analysis using this cutoff value did not reveal any significant differences. Previously, a relationship between high ACSS2 levels and a greater liver tumor burden was shown in mouse models but not in humans^5^. Therefore, we reanalyzed the patient data with the best risk separation approach^18^ and found a highly significant and large association. This approach enables the discovery of a disproportionate but clinically useful stratification that might have been missed with an equal proportion, that is, a 50:50 group separation. There was a subset of patients in the low-ACSS2 subgroup belonging to the lower 13th percentile of ACSS2 expression; these patients were classified as the “very low” ACSS2 subgroup (13VLA group). Compared to those in the remaining 87th percentile group, the prognosis was particularly poor for all available survival-related variables (Fig. 1f): overall survival (OS), disease-specific survival (DSS), the DFI, and the PFI. In comparison, acetyl-CoA synthetase 1 (ACSS1) levels, which were suggested to be elevated in a high malignancy group in a previous report (approximately 30% of a cohort)^10^, did not differ between these two groups (Supplementary Fig. 2). These results were confirmed with another independent large cohort (GSE76427), in which patients in the lower 17^th^ percentile exhibited markedly shorter OS and relapse-free survival (Fig. 1g). Overall, these findings confirm the link between lower ACSS2 expression and more severe malignancy in liver cancer patients.

### PET-CT imaging of low- and high-grade liver cancer patients

**Fig. 2:**
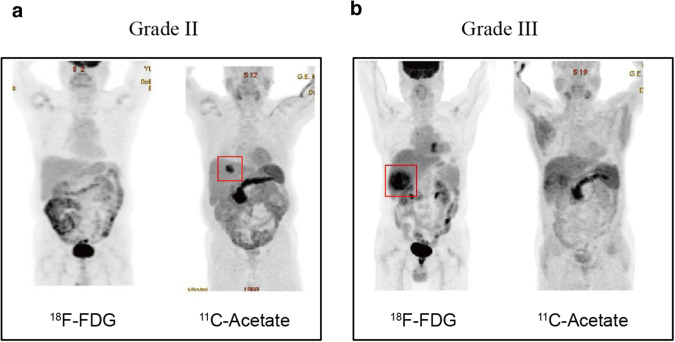
Positron emission tomography/computed tomography (PET-CT) imaging of low- and high-grade liver cancer patients. **a** Representative PET‒CT images of a patient with low-grade (grade II) liver cancer. **b** Representative PET-CT images of high-grade (grade III) liver cancer patients, each of whom was monitored with both ^18^F-fluorodeoxyglucose (^18^F-FDG) and ^11^C-acetate. (See Supplementary Fig. 3a, b for data from eight additional patients.) Cancer regions are marked with red boxes.

Acetate metabolism through ACSS2 has been reported to be important in carcinogenesis^13^. Therefore, to directly assess the metabolite-malignancy relationship in living human patients, PET/CT data with ^11^C-acetate and ^18^F-FDG and tumor grade were analyzed. A much greater ^11^C-acetate level with little ^18^F-FDG uptake was found in lower-grade (grade II) patients, whereas little ^11^C-acetate but prominent ^18^F-FDG uptake was noted in higher-grade hepatocellular carcinoma (grade III) patients (Fig. 2a, b; Supplementary Fig. 3 for the other eight patients). Metabolic stress owing to low blood supply, such as hypoxia or nutrient depletion, would have exerted similar effects on the uptake of acetate and the uptake of FDG. However, the selective uptake of either acetate or glucose in the imaging results seemed to exclude low blood supply as a condition for acetate uptake. Therefore, acetate uptake in lower-grade liver cancers appears to be an inherent property. These data confirm the relationship between high acetate uptake and lower malignancy, independent of metabolic stress.

### Acetate uptake and its relationship with cancer cell growth under varying glucose concentrations

**Fig. 3:**
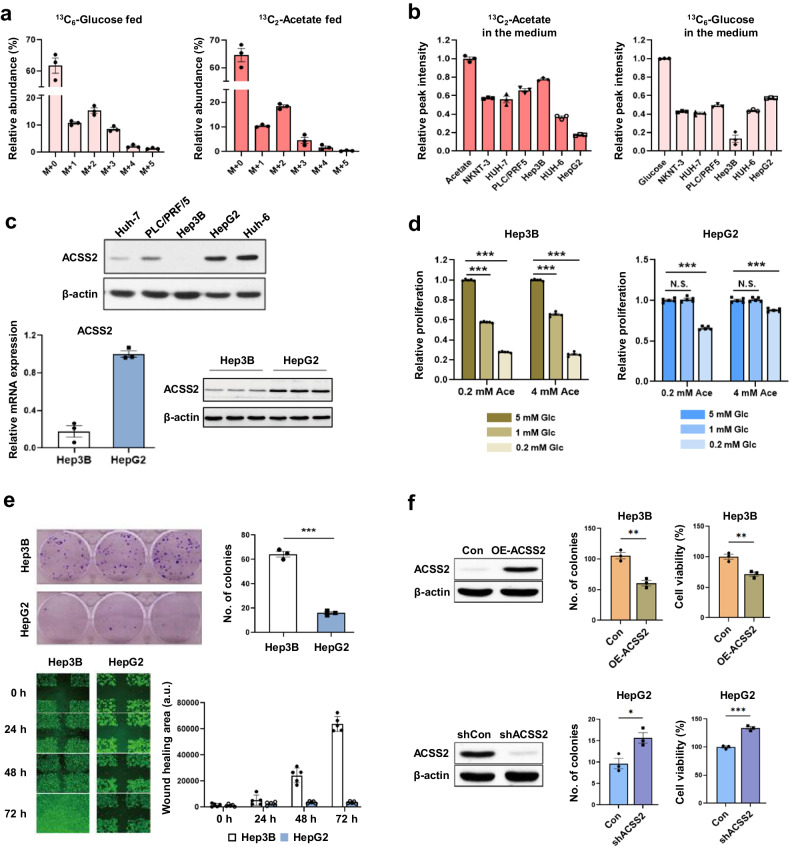
Acetate uptake in normal mouse liver tissue and cancer cells and its relationship with cell growth. **a** Glutamate isotopomer distribution in normal liver tissue of mice orally administered U-^13^C_6_-glucose (left) or 1,2-^13^C_2_-acetate (right) (both 5 g/kg), as measured via liquid chromatography–mass spectrometry (LC–MS). The sampling times for the tracers differ to reflect the kinetic differences in absorption between the two tracers. **b** Uptake of 1,2-^13^C_2_-acetate (left, 0.2 mM) or U-^13^C_6_-glucose (right, 5 mM) by various liver cell lines measured by nuclear magnetic resonance (NMR) intensities of the remaining tracers in the media compared with their initial intensities (leftmost for each). **c** ACSS2 expression in various liver cancer cells. Western blot analysis of ACSS2 expression in Huh-7, PLC/PRF/5, Hep3B, HepG2, and Huh-6 cells and real-time quantitative polymerase chain reaction (qRT–PCR) analysis of ACSS2 expression in Hep3B and HepG2 cells. **d** Liver cancer cell growth was measured by a colorimetric cell viability assay in the presence of the indicated concentrations of acetate and glucose. **e** Clonogenic and scratch wound healing assays were performed for Hep3B and HepG2 cells. **f** Cell survival of shACSS2 HepG2 and OE-ACSS2 Hep3B cells determined by clonogenic assay after 14 days and cell proliferation of shACSS2 HepG2 and OE-ACSS2 Hep3B cells after 48 h. N.S.: not significant; *p < 0.05, **p < 0.01, **p < 0.001 (*n* = 3) according to Student’s *t* test with standard deviations.

Despite reports suggesting that acetate uptake occurs primarily during glucose depletion or hypoxia^5,8^, such conditions also limit acetate usage. This led us to hypothesize that, if tissues use acetate, nutrient depletion may not always be a prerequisite. Therefore, we first tested whether acetate can be used in normal liver tissues (in which nutrients and oxygen are replete) and compared the results with those of glucose utilization. Mice were orally administered the same amount of ^13^C-acetate and ^13^C-glucose, and their liver tissues were analyzed for isotope incorporation into glutamate^7^. The ratios of the ^13^C-labeled glutamate isotopomers and M + 2 isotopomer, formed from the first cycle of acetate incorporation^7^, were similar in both groups (Fig. 3a). These data showed that the liver can utilize acetate carbons as efficiently as glucose carbons under normal conditions. In addition, a normal liver should express high levels of ACSS2 for acetate utilization, as shown in Fig. 1. Acetate uptake by cancer cells was evaluated in various liver cell lines in the presence of physiological concentrations of acetate and glucose. Among the cells, the most efficient acetate uptake, in HepG2 cells, was approximately fourfold greater than that in Hep3B cells, whereas the Hep3B cells consumed much more glucose than the HepG2 cells did (Fig. 3b). Notably, the expression levels of ACSS2, which is vital for acetate utilization^5,7,19^, were significantly high in HepG2 cells but lowest in Hep3B cells (Fig. 3c). These data showed that liver cancer cells with high ACSS2 expression can efficiently uptake acetate, even under glucose-free conditions.

To test whether differential acetate uptake affects cell phenotypes, we measured cell growth. In the presence of 0.2 mM acetate, lowering the glucose concentration from 5 mM to 1 mM significantly inhibited the growth of Hep3B cells but not HepG2 cells with low ACSS2 expression (Fig. 3d). An even lower concentration of glucose (0.2 mM) further suppressed the growth of Hep3B cells, but this effect was significantly reduced in HepG2 cells treated with acetate. Under these conditions, a high concentration of acetate (4 mM) significantly rescued the growth of the HepG2 cells but not the Hep3B cells. In addition, clonogenic and wound healing assays revealed that cell growth and migration were greater in ACSS2-low Hep3B cells than in ACSS2-high HepG2 cells **(**Fig. 3e). However, these effects were reversed when ACSS2 was knocked down or overexpressed in HepG2 and Hep3B cells, respectively (Fig. 3f, Supplementary Fig. 4). Overall, acetate uptake under normal physiological conditions seems to be an inherent property of liver tissue, and there is heterogeneity in acetate uptake among liver cancer cells based on the expression of ACSS2, which in turn affects liver cancer cell growth. Therefore, subsequent experiments on the heterogeneity of acetate metabolism and related cell physiology in liver cancer were performed using high-ACSS2 HepG2 cells and low-ACSS2 Hep3B cells.

### Differential metabolic fates of acetate and differential gene expression in low-ACSS2 and high-ACSS2 cells

**Fig. 4:**
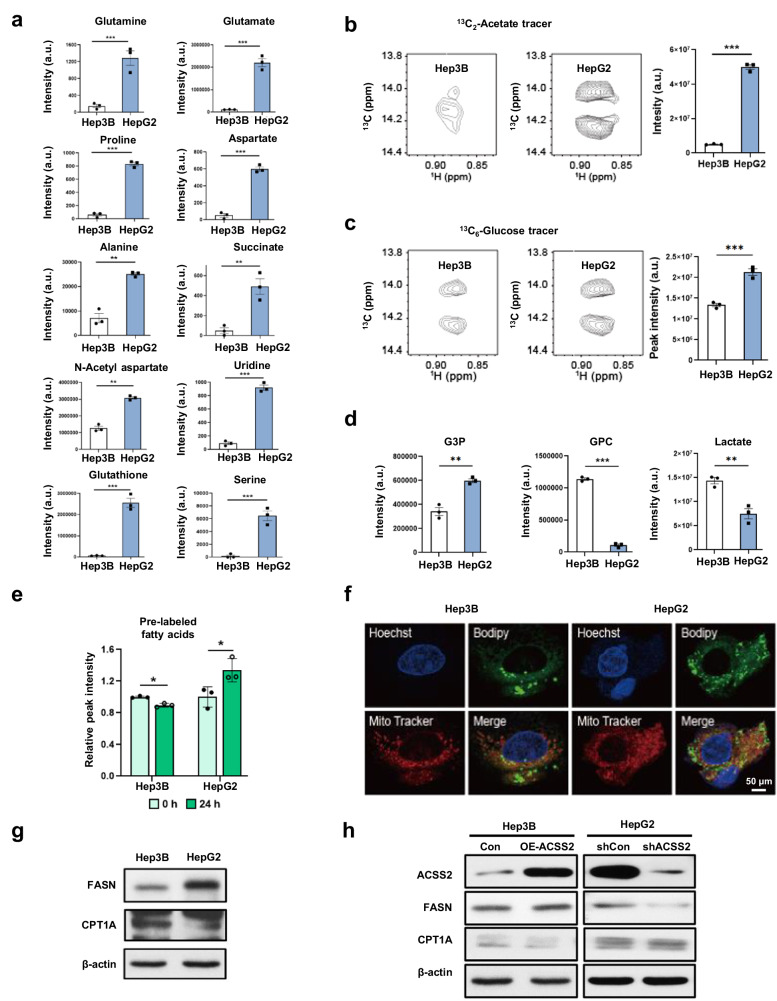
Different metabolic fates of acetate in high- and low-ACSS2 cells. **a** Isotopic incorporation of 0.2 mM 1,2-^13^C_2_-acetate into various metabolites in Hep3B and HepG2 cells, as measured with ^13^C-^13^C *J*-split peaks in 2D NMR spectra. **b** and **c** Fatty acid de novo synthesis by 0.2 mM 1,2-^13^C_2_-acetate or 5 mM U-^13^C_6_-glucose in Hep3B and HepG2 cells as measured by the intensities of omega methyl splitting from high-resolution NMR spectra (left two spectra). Peak intensity averages (right bar graphs). **d** Isotopic incorporation of 5 mM U-^13^C_6_-glucose. **e** Changes in prelabeled ^13^C ω-carbon fatty acids 24 h after removing the ^13^C carbon source in the media. **f** Lipid droplet levels (Bodipy FL C_12_; green) measured via confocal microscopy. Nuclei (Hoechst 33342; purple) and mitochondria (MitoTracker Red CMXRos; red). **g**, **h** Expression of FASN and CPT1A was detected in HepG2, Hep3B, shACSS2, and OE-ACSS2 Hep3B cells by western blotting. *p < 0.05, **p < 0.01, and **p < 0.001 (*n* = 3).

Next, for the high- and low-ACSS2 cells, the incorporation of ^13^C-acetate into cellular metabolites was measured using high-resolution 2D HSQC NMR (Supplementary Fig. 5). HepG2 cells utilize ^13^C-acetate in diverse biosynthetic pathways, leading to its incorporation of various amino acids, the tricarboxylic acid (TCA) intermediate succinate, the acetylated amino acid N-acetyl aspartate, and even the nucleic acid base uridine (Fig. 4a). In this study, the labeling of TCA intermediates using ACSS2 was consistent with the methods used in previous studies^5,7^. In comparison, the Hep3B cells did not show appreciable incorporation of most of these metabolites, which is consistent with the negligible effects of acetate on Hep3B growth. Acetate incorporation was also measured in hydrophobic lipids using ω-methyl NMR peak splitting^19^. Compared with Hep3B cells, HepG2 cells exhibited greater de novo fatty acid synthesis from ^13^C-acetate (Fig. 4b) and, interestingly, even from ^13^C-glucose (Fig. 4c). Consistently, the ^13^C incorporation from ^13^C-glucose to glycerol 3-phosphate, a substrate of the rate-limiting enzyme of lipid synthesis (i.e., glycerol-3-phosphate acyltransferase (GPAT)), was greater in HepG2 cells than in control cells (Fig. 4d). Conversely, glycerophosphocholine, a final product of catabolic glycerophospholipid lipases, was strongly associated with ^13^C-glucose in Hep3B cells. The greater incorporation of ^13^C-glucose into lactate suggested more active catabolic glycolysis in the Hep3B cells. This greater lipid catabolism was also confirmed by the greater loss of the prelabeled ^13^C ω-carbon fatty acid signal in the Hep3B cells than in the HepG2 cells upon removal of the ^13^C carbon source in the media (Fig. 4e). Further measurement of lipid droplets by fluorescence showed a significantly greater lipid content in the HepG2 cells (Fig. 4f). To validate the greater fatty acid synthesis capacity of HepG2 cells than of Hep3B cells, we evaluated the expression of FASN, a key rate-limiting enzyme in fatty acid synthesis, and carnitine palmitoyl transferase 1 (CPT1), a rate-limiting enzyme involved in mitochondrial fatty acid oxidation. As a result, FASN expression was greater in HepG2 cells than in Hep3B cells, whereas CPT1A expression was greater in Hep3B cells than in HepG2 cells (Fig. 4g). These results were confirmed in shACSS2 HepG2 and OE-ACSS2 Hep3B cells (Fig. 4h). These results suggest that high-ACSS2 HepG2 cells have a greater anabolic capacity when using both acetate and glucose, whereas Hep3B cells are more catabolic, especially in terms of lipid metabolism.

To determine whether the above differential metabolic characteristics were reflected at the gene expression level, the transcriptomic profiles of the cells were analyzed using the GSE21955 dataset. The GSEA results for the metabolic genes showed that the HepG2 cells were significantly enriched in the anabolic pathways for lipids and amino acids and in the pathways for a master regulator of lipid synthesis (a sterol regulatory-element binding protein) (Supplementary Fig. 6). In addition, the expression of several key genes in diverse lipid biosynthetic pathways, such as *ACACA, FASN*, and *GPAM*, was consistently upregulated in HepG2 cells (Supplementary Fig. 7a, b). These results support the idea that high-ACSS2 HepG2 cells with high levels of acetate uptake have more anabolic effects.

### Modulation of ACSS2 activity affects the metabolic phenotypes of liver cancer cells treated with high acetate concentrations

**Fig. 5:**
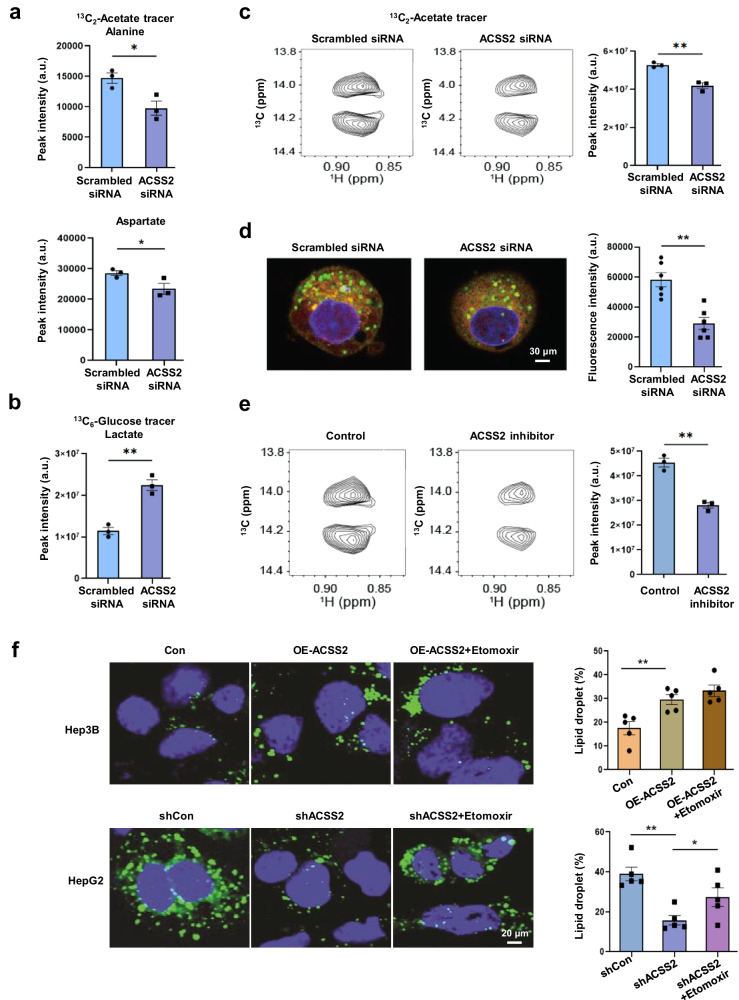
Modulation of ACSS2 affects the phenotypes of HepG2 cells with high ACSS2 expression. **a** ^13^C-alanine and ^13^C-aspartate levels in HepG2 cells after ACSS2 knockdown were measured with 1,2-13C2-acetate, as described in Fig. 4a. **b** ^13^C-labeled lactate from 5 mM U-^13^C_6_-glucose in HepG2 cells upon ACSS2 knockdown, as measured by the intensities of omega methyl splitting from high-resolution NMR spectra. **c**, **d** Inhibition of fatty acid synthesis in HepG2 cells by ACSS2 knockdown, as measured in Fig. 4b, and inhibition of lipid droplet formation in HepG2 cells by ACSS2 knockdown, as shown in Fig. 4f. Bodipy FL C12; green, Hoechst 33342; purple, MitoTracker Red CMXRos; red. **e** Inhibition of fatty acid synthesis from 0.2 mM 1,2-^13^C_2_-acetate in HepG2 cells by an ACSS2 inhibitor (1-(2,3-di(thiophen-2-yl)quinoxalin-6-yl)-3-(2-methoxyethyl)urea, 10 μM). **f** Lipid droplet counts (Bodipy FL C_12_; green) measured via confocal microscopy after etomoxir treatment (150 µM, 24 h) in shACSS2 HepG2 and OE-ACSS2 HepG3B cells. *p < 0.05, **p < 0.01 (*n* = 3).

To establish the causal relationship between ACSS2 and the anabolic characteristics of HepG2 cells, we modulated ACSS2 expression. siRNA-mediated knockdown of ACSS2 led to a decrease in the incorporation of ^13^C-acetate into metabolites such as alanine and aspartate, which exhibited greater incorporation in HepG2 cells (Fig. 5a). Interestingly, ACSS2 knockdown increased lactate production from ^13^C-glucose in HepG2 cells (Fig. 5b), suggesting that ACSS2 downregulation results in more Hep3B-like cells, i.e., more glycolytic cells. Importantly, the knockdown of this gene reduced the de novo synthesis of fatty acids from ^13^C-acetate and the lipid droplet content in HepG2 cells (Fig. 5c, d). The effect of ACSS2 modulation was also tested with an ACSS2-specific inhibitor (1-(2,3-di(thiophen-2-yl)quinoxalin-6-yl)-3-(2-methoxyethyl)urea)^5^, which decreased de novo fatty acid synthesis in HepG2 cells (Fig. 5e). These results were confirmed by the reduction in lipid accumulation in shACSS2 HepG2 cells and by the restoration of lipid content by etomoxir, a lipid oxidation inhibitor (Fig. 5f). However, etomoxir, which blocks beta-oxidation, did not significantly increase lipid deposition in Hep3B cells treated with OE-ACSS2. Taken together, these findings suggest that artificial ACSS2 overexpression in Hep3B cells may lead to an increase in the concentration of malonyl CoA, a very potent inhibitor of CPT1, a key component of fatty acid oxidation, resulting in decreased etomoxir efficacy. Another reason may be that the acetyl-CoA from ACSS2 may be preferentially directed toward lipid synthesis, which could explain the association between high ACSS2 expression and lipid anabolism. Taken together, these data show that ACSS2 is involved in anabolic lipid metabolism in high-ACSS2 liver cancer cells and that the inhibition of ACSS2 can increase glycolysis.

### High-ACSS2 liver cancer leads to the formation of anabolic and less malignant tumors in vivo

**Fig. 6:**
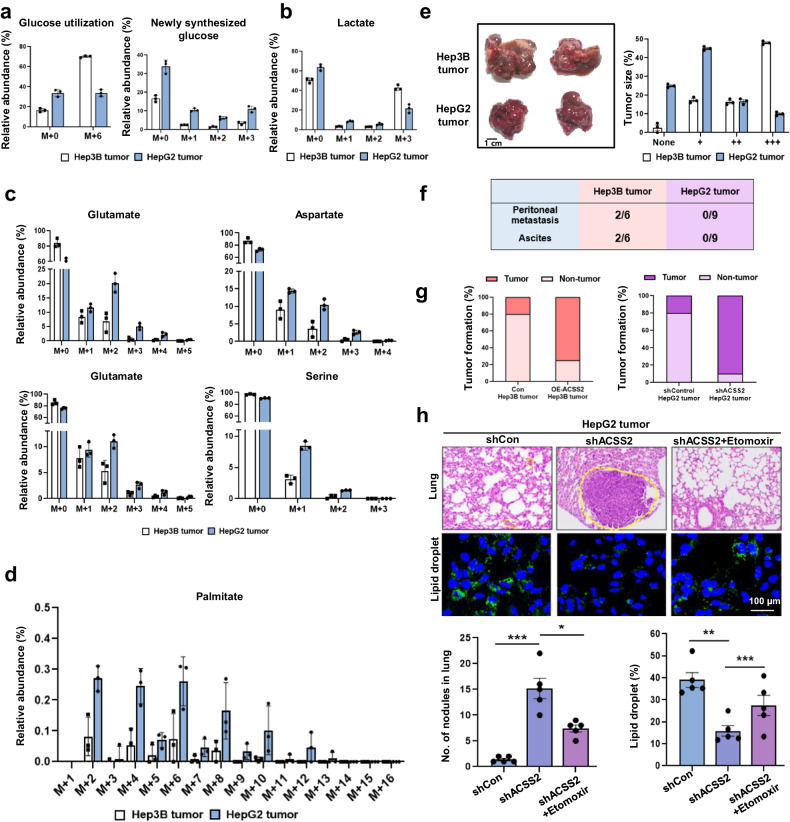
High-ACSS2 cells forms anabolic and less malignant tumors in HepG2 and Hep3B orthotopic mouse xenograft models. **a** U-^13^C_6_-glucose utilization (left) and newly synthesized glucose (right) from liver cancer tissues of mice orally administered 5 g/kg U-^13^C_6_-glucose, as measured via LC‒MS. **b** ^13^C-lactate generation from U-^13^C_6_-glucose in liver cancer tissues. **c** Isotopic incorporation of various metabolites from the livers of mice orally administered 1,2-^13^C_2_-acetate (3 g/kg), as measured via LC‒MS. **d** Isotopic incorporation of 1,2-^13^C_2_-acetate into palmitate in liver tissues, as measured via LC‒MS. **e** Representative mouse liver tumors (left, *n* = 8–9) and the tumor size distribution (right) after 2 months (number of + signs: overall visible sizes). **f** The frequency of peritoneal or ascites metastasis. **g** Tumor occurrence rates in orthotopic mice injected with shControl/shACSS2-treated HepG2 cells or OE-control/OE-Hep3B cells. **h** Lung metastasis and lipid droplets in shACSS2 HepG2 orthotopic mouse models after etomoxir treatment for 4 weeks (50 mg/kg, every other day, IP). Lungs were excised and processed for H&E staining following metastatic scoring. The dotted lines indicate metastatic nodules and the metastatic area. *p < 0.05, ***p < 0.001 (*n* = 3).

To validate the above results in vivo, isotope tracing with ^13^C-acetate or ^13^C-glucose and phenotypic characterization were performed on orthotopic liver cancer mouse models using HepG2 or Hep3B cells. After U-^13^C-glucose administration, the M + 6 isotopomer ratio of glucose was much greater in the Hep3B tumors than in the control tumors, indicating increased glucose utilization (Fig. 6a). However, the M + 1, +2, and +3 isotopomers of glucose, formed only through the resynthesis of glucose (gluconeogenesis), were higher in the HepG2 tumors, indicating that anabolic gluconeogenesis was more active in HepG2 tumors. In contrast, the opposite catabolic glycolytic activity was greater in Hep3B tumors, as estimated by M + 3 lactate production from ^13^C-glucose (Fig. 6b). Following ^13^C-acetate administration, a HepG2 tumor exhibited enhanced incorporation of ^13^C atoms into various amino acids (Fig. 6c) and glucose (Supplementary Fig. 8). The HepG2 tumors again showed greater incorporation of ^13^C-acetate into fatty acids in vivo, as shown by the high-molecular-weight isotopomers of palmitate (Fig. 6d). As the serum concentrations of ^13^C-acetate and ^13^C-glucose were not different between the HepG2 and Hep3B tumors (Supplementary Fig. 9), the differences above reflect the metabolic differences in the tumor tissues rather than the serum availability of the nutrients.

Regarding phenotypic malignancy, the tumor frequency, tumor size (Fig. 6e), and frequency of metastasis to either the peritoneum or ascites (Fig. 6f) were all lower or smaller in HepG2 tumors, indicating that liver cancer cells that uptake acetate are less tumorigenic and form fewer or smaller malignant tumors. In addition, according to a hallmark gene set analysis, a lower epithelial mesenchymal transition (EMT) is the top biological process differentiating high-ACSS2 HepG2 cells from low-ACSS2 Hep3B cells (Supplementary Fig. 10a)^20^. To further confirm whether ACSS2 affects the regulation of EMT, we measured the expression levels of E-cadherin (epithelial marker) and N-cadherin (mesenchymal marker) and of EMT transcription factors, including Zeb1, Snail, and Twist, in shACSS2 HepG2 and OE-ACSS2 Hep3B cells. Consistent with the results of the gene set analysis, high-ACSS2 decreased EMT signaling in OE-ACSS2 Hep3B cells, whereas low-ACSS2 increased this signaling in shACSS2 HepG2 cells. In particular, among the EMT transcription factors, ACSS2 was found to be regulated by Zeb1 (Supplementary Fig. 10b). In addition, as mentioned above, the growth of the HepG2 cells was much slower than that of the Hep3B cells; however, the shACSS2-treated HepG2 cells exhibited an enhanced growth rate (Fig. 3e, f). This finding suggested that shACSS2-treated HepG2 cells exhibit more Hep3B-like cells, consistent with the greater glycolytic phenotype of HepG2 cells. To confirm these in vivo results, we transplanted shACSS2-overexpressing HepG2 cells into orthotopic mouse models. The results showed that the tumor occurrence rate was much higher in orthotopic liver cancer mice injected with shACSS2-expressing HepG2 cells (6 out of 7 = 85.7%) than in the control group (1 out of 8 = 12.5%) (Fig. 6g, Supplementary Fig. 11a), consistent with the in vitro results showing faster growth of shACSS2-expressing HepG2 cells (Fig. 3e, f). In contrast to the shACSS2 HepG2 liver cancer mouse model, the tumor occurrence rate was much lower in the orthotopic liver cancer mouse model generated from OE-ACSS2 Hep3B cells (2 out of 8 = 25%) than in the control group (8 out of 10 = 80%). Together with the prevention of lung metastasis, these decreases in lipid content were reversed by etomoxir in shACSS2 HepG2 tumors (Fig. 6h). In addition, shACSS2-treated HepG2 tumors exhibited a reduction in lipid content and high lung metastasis. Furthermore, higher lactate levels in shACSS2 HepG2 tumors indicated a higher degree of glycolysis (Supplementary Fig. 11b). As increased growth and enhanced glycolytic activity are characteristics of highly malignant human tumors, these data confirm that acetate-use-related tumors with increased ACSS2 levels exhibit anabolic characteristics and decreased malignancy.

### Metabolic characteristics of high- and low-ACSS2 human liver cancer

The metabolic characteristics of the high- and low-ACSS2 groups were investigated based on their gene expression profiles. A GSEA of the TCGA cohort showed that the expression of genes related to anabolic lipid metabolism was significantly elevated in the high-ACSS2 patient cohort defined by the 2352 RSEM cutoff value (Supplementary Fig. 12). At the individual gene level, the key genes in the lipid anabolic pathway, i.e., *SREBF1, SREBF2, HMGCS1, HMGCS2, FASN, ACACA, ACLY*, and *GPAM*, were consistently upregulated in the high-ACSS2 subgroup (Supplementary Fig. 12b). Similar results were also observed between the 13VLA group and the other groups (Supplementary Fig. 13a, b). Interestingly, for the 13VLA group, the expression of key genes in glycolytic pathways, such as *LDH*, *HK*, and *PFK*, was significantly upregulated relative to that in the other groups, indicating high glucose catabolism (Supplementary Fig. 13c); these findings are consistent with the cell and animal results. Previously, hypoxia was suggested to induce ACSS expression, increased acetate usage, and increased malignancy in several cancers, including liver cancer^8,9^. In contrast, our analysis revealed significant negative correlations between ACSS2 expression and a well-established hypoxia score (Supplementary Fig. 13d)^21^, suggesting that high ACSS2 expression is associated with decreased hypoxia. Indeed, the hypoxia score was significantly greater in the 13VLA group than in the other groups (Supplementary Fig. 13e).

Next, the 13VLA group and the others were analyzed for DNA promoter methylation, as this has important implications for liver cancer carcinogenesis and prognosis^22,23^. Strikingly, the sites in ACSS2 and FASN were among the top 20 (approximately 390,000) sites that were differentially methylated between the two groups (Supplementary Fig. 13f). Specifically, the site at the CpG shore of the ACSS2 promoter (for the cg09801828 probe) exhibited significantly greater methylation in the 13VLA group than in the other groups (Supplementary Fig. 13g), suggesting a role for DNA methylation in ACSS2 suppression. Overall, these patient data analyses showed that high ACSS2 expression is associated with lipid anabolic characteristics, reduced glycolysis, and decreased hypoxia and malignancy, thereby confirming our cell- and animal-level results.

## Discussion

Despite prominent studies suggesting that acetate usage is limited to nutrient-depleted or hypoxic conditions^5,8^ and links between high ACSS2/acetate usage and enhanced cancer malignancy^7,19^, such generalizations may require caution. Conceptually, nutrient depletion occurs owing to a low blood supply, which may limit acetate availability. Indeed, our cell, animal, and patient results consistently showed that high acetate uptake/ACSS2 expression can occur in glucose- or nutrient-replete conditions in lower-grade liver cancer cells or in tumors that feature reduced hypoxia. In addition, hypoxia generally activates glycolytic activity and is also associated with increased malignancy^11,12^. Increased glycolytic metabolism may reduce acetate usage; therefore, the link between acetate uptake and increased malignancy may not be universal. In colorectal cancer patients, lower acetate uptake is attributed to greater malignancy^24^. ACSS2 levels are generally high in normal human liver tissues^25,26^, and it has been noted that ACSS2 is maintained in high-ACSS2 liver cancer cells but is lost in low-ACSS2 cancer cells^5^. Based on our results and the established roles of ACSS2 in acetate usage^7,27,28^, ACSS2-driven high acetate uptake seems to be an inherent property of a normal liver that may be only slightly lost (if at all) in low-malignancy liver cancer but largely lost in high-malignancy liver cancer.

In a bioinformatics-focused study, a high acetate uptake through ACSS1 under hypoxia was proposed for an iHCC3 group with shorter patient survival, and hence, higher malignancy^10^. Unfortunately, the effects of hypoxia were not explicitly addressed for ACSS1 or ACSS2 in their experimental model HepG2 cells. Interestingly, we found a high ACSS2 level in the iHCC1 subgroup, which had the lowest malignancy and longest survival^10^, consistent with our overall results. Another study performed a knockdown of ACSS2 in liver cancer cells but in a highly malignant cell line with lower ACSS2 levels (MHCC97H) rather than a lower malignant cell line with higher ACSS2 levels (MHCC97L)^29^, making it difficult to compare the results with our findings. According to our results, the knockdown of ACSS2 in HepG2 cells induced the increase in glycolytic lactate production. This was also confirmed in vivo tumors, and 13VLA patients exhibited upregulation of glycolytic genes. Therefore, ACSS2 loss appears to occur concomitantly with enhanced glycolysis. This may render cancer cells more malignant and more likely to be viable under hypoxic conditions and with the Warburg phenotype; these findings are consistent with our PET-CT results. It is tempting to speculate that ACSS2 may have an inhibitory effect on glycolysis, providing an interesting topic for future studies.

Our results have important translational implications. First, PET-CT-detectable metabotypes of ACSS2-driven acetate uptake may be noninvasive biomarkers for lower-malignancy liver cancers. The use of both ^11^C-acetate and ^18^F-FDG may overcome the unacceptably high false-negative rate (40–50%) of single ^18^F-FDG imaging^14^ and should also help discriminate between acetate-using (acetophilic) or glycolytic (glucophilic) liver cancers. In comparison, previous biomarkers based on transcriptomics or genetics require invasive biopsies^23,30,31^. The higher ^11^C-acetate signal in the tumor region relative to that in the normal liver background in patients with low-grade liver cancer may be due to multiple factors. One possibility could be the rapid oxidation and elimination of acetate to CO_2_ in normal tissue, but the exact reasons may require further study. Second, for patient stratification, the metabolic patterns of the 13VLA subgroup, which has a particularly poor prognosis, were not apparent in earlier stratification studies. Hoshida et al. reported that the most malignant tumor group (Supplementary Fig. 1) constituted 25–33% of the cohort^30^. Two major subtypes (proliferation and nonproliferation classes), each accounting for approximately 50%, were identified by analyzing many previous studies^31^. In a omics-based study^23^, approximately 33% of the cohort (iClust1 group) were found to have high-grade tumors with poor prognosis. According to a bioinformatics study, the iHCC3 cohort (comprising approximately 28% of the cohort) exhibited shorter survival times and high glycolytic and fatty acid synthesis-related gene expression^10^. Therefore, the 13VLA subgroup seems to constitute a smaller proportion (approximately 13%) of patients with distinct metabolic characteristics and, thus, is proposed as a new subset with the poorest prognosis among the poor prognosis groups.

**Fig. 7:**
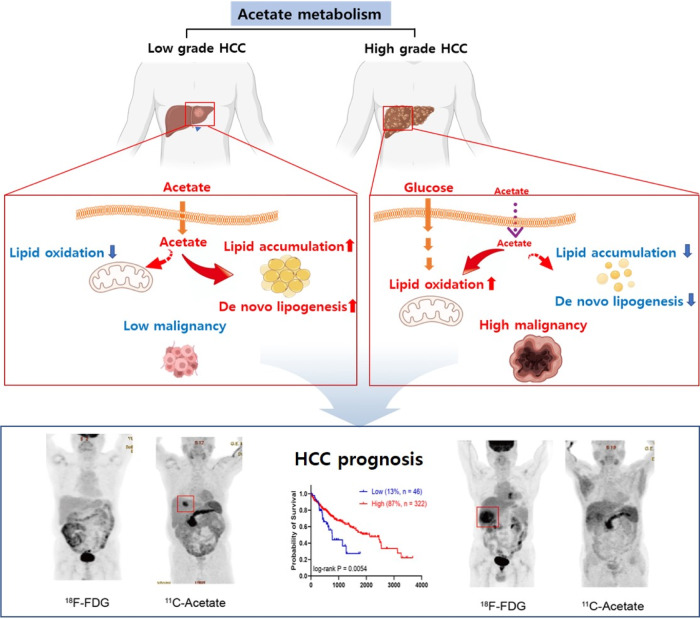
Role of ACSS2 in acetate metabolism of HCC. Summary of the mechanism through which ACSS2 affects liver cancer prognosis through the switch of acetate metabolism.

In conclusion, we suggest that there is a link between high acetate usage, anabolic characteristics, and low malignancy in liver cancer patients according to comprehensive studies on cells, animals, and humans. We also identified a patient group with a particularly poor prognosis due to very low ACSS2 expression and unfavorable glycolytic characteristics. Our results may help to stratify patients with liver cancer, thus enabling the selection of the optimum treatment option(s) (Fig. 7).

### Supplementary information

Supplemental information

Supplementary Table 1

## Data Availability

The TCGA mRNA expression data, beta values, and survival information used in the present study are available at https://gdc.cancer.gov/about-data/publications/pancanatlas. The tumor grade information was obtained from the TCGA GDC portal (http://portal.gdc.cancer.gov/). Disease-free status information and hypoxia scores were downloaded from the cBioPortal (www.cbioportal.org). The Gene Expression Omnibus (GEO) datasets used in this study were GSE21955 and GSE76427.
