# Supplementary material for: Acetyl-CoA synthetase 2 contributes to a better prognosis for liver cancer by switching acetate-glucose metabolism
Source: Exp Mol Med. 2024 Mar 25;56(3):721–33. doi: 10.1038/s12276-024-01185-3 (PMC10984961; doi:10.1038/s12276-024-01185-3)
Supplement: Supplementary file 1 — Supplemental information [file 12276_2024_1185_MOESM1_ESM.pdf]

Supplementary Figures

Acetyl-CoA synthetase 2 contributes to a better prognosis for liver cancer by switching acetate-glucose metabolism

Kyung Hee Jung[1,*, #], Sujin Lee[2,*], Han Sun Kim[2,*], Jin-Mo Kim[2,*], Yun Ji Lee[1], Min Seok Park[1], Myeong-Seong Seo[1], Misu Lee[3], Mijin Yun[4,#], Sunghyouk Park[2, #], and Soon-Sun Hong[1,#]

Contests

1. **Supplementary Figs. 1-13**

Supplementary Fig. 1

a

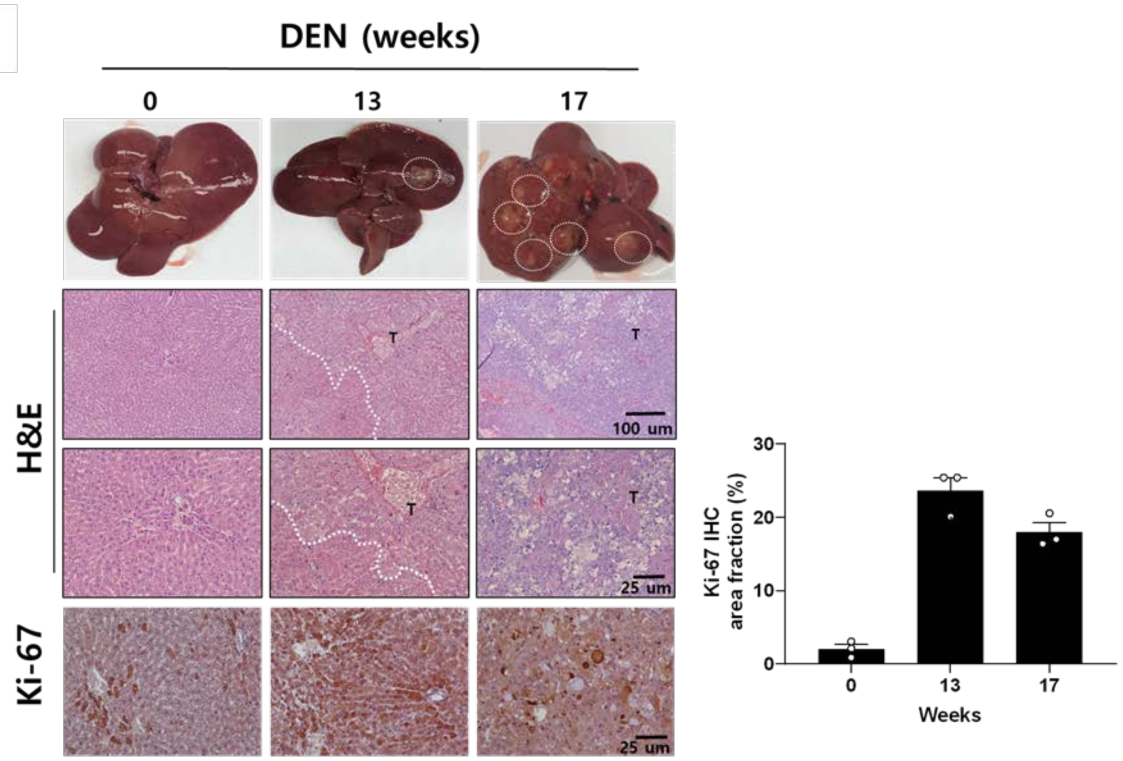

b

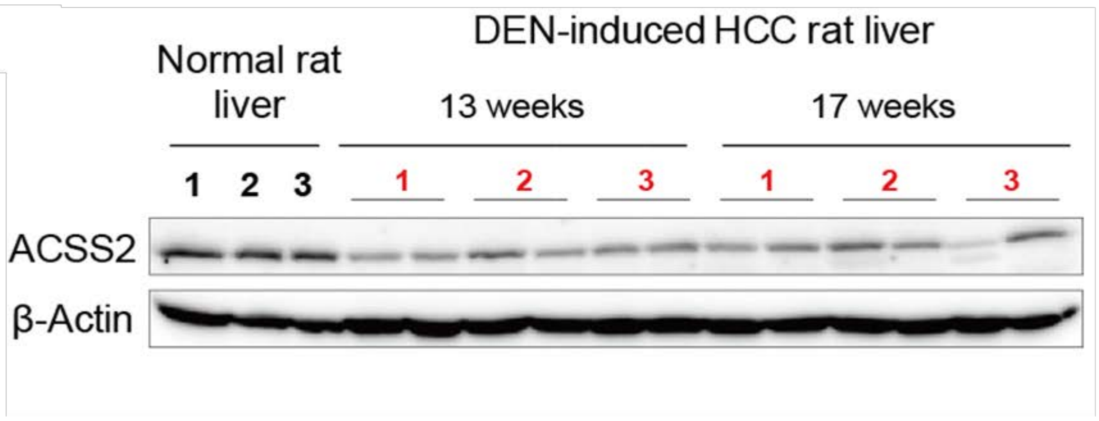

Supplementary Fig. 2

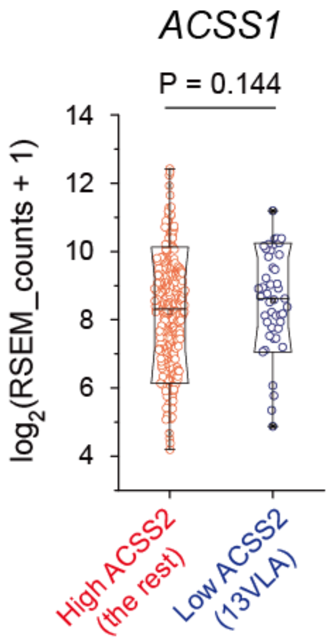

Supplementary Fig. 3

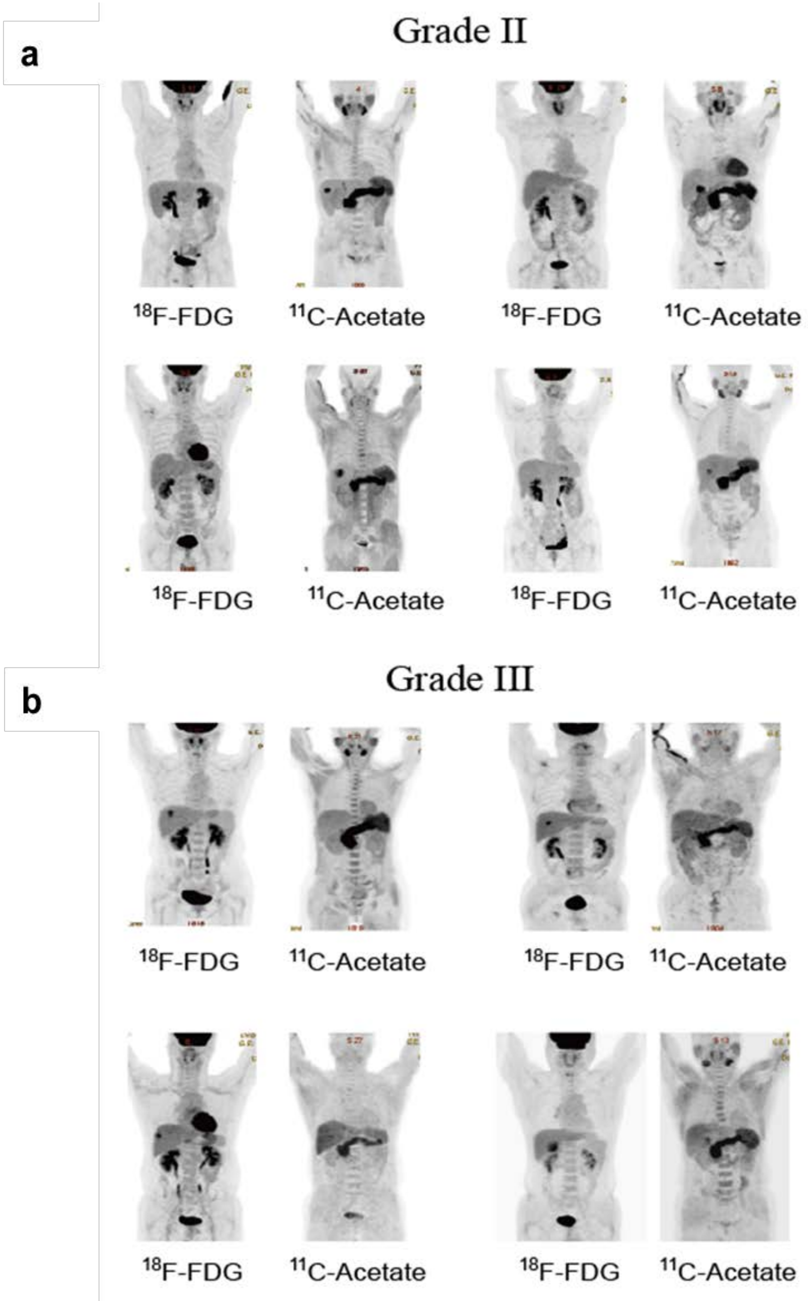

Supplementary Fig. 4

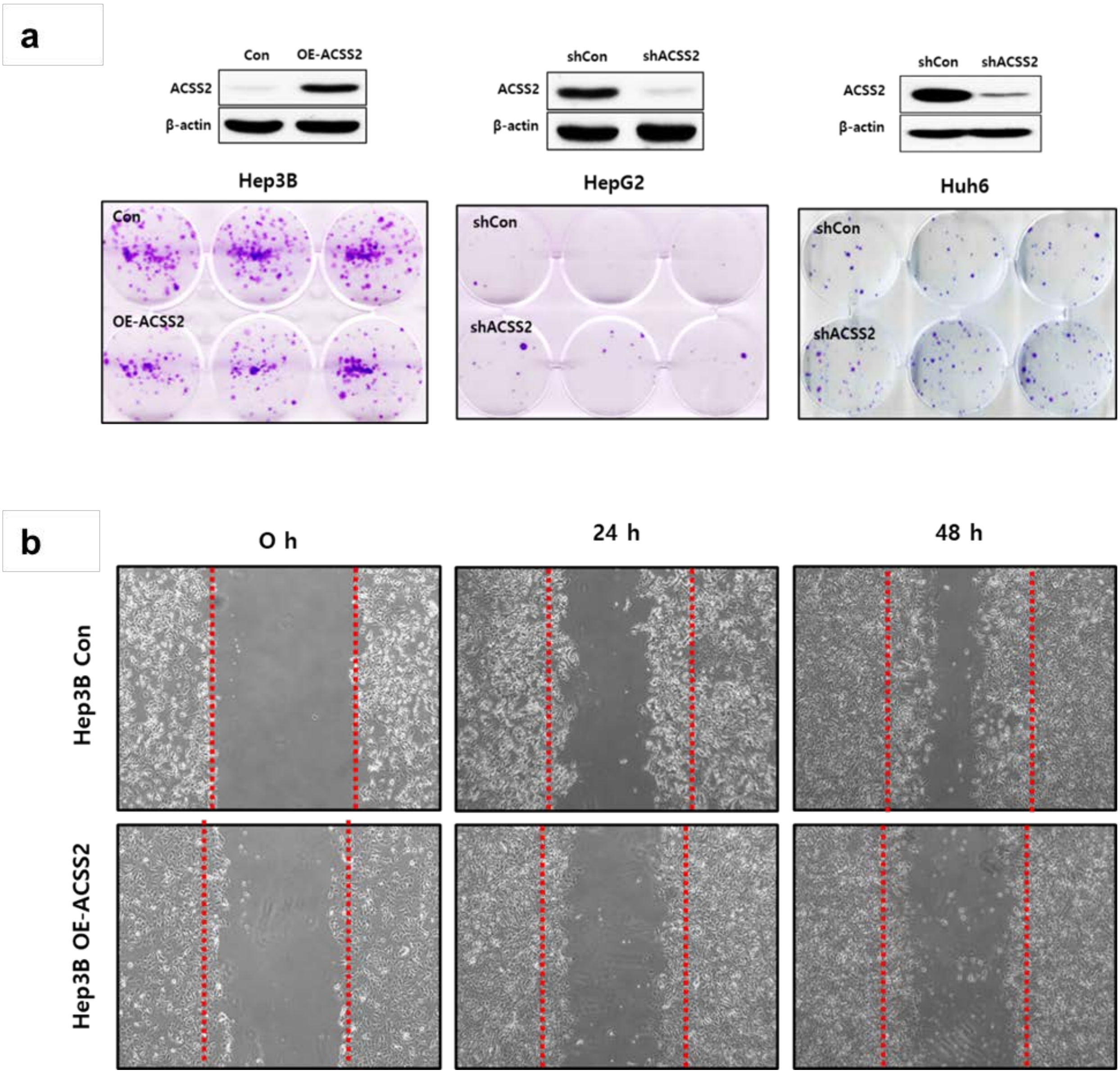

Supplementary Fig. 5

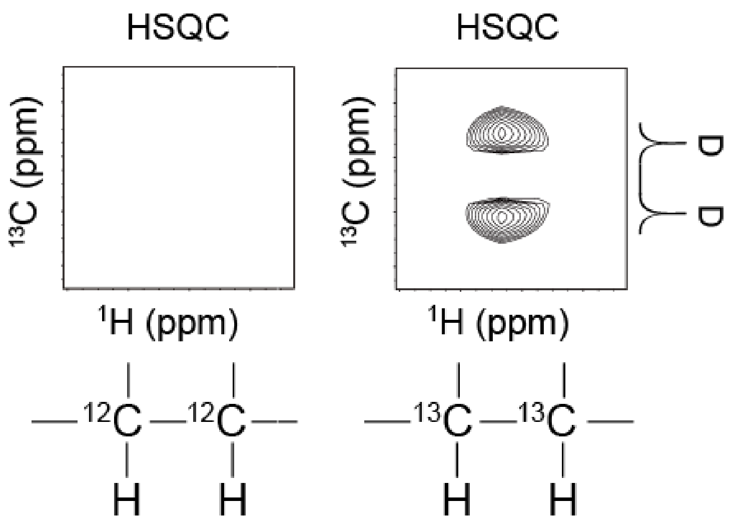

Supplementary Fig. 6

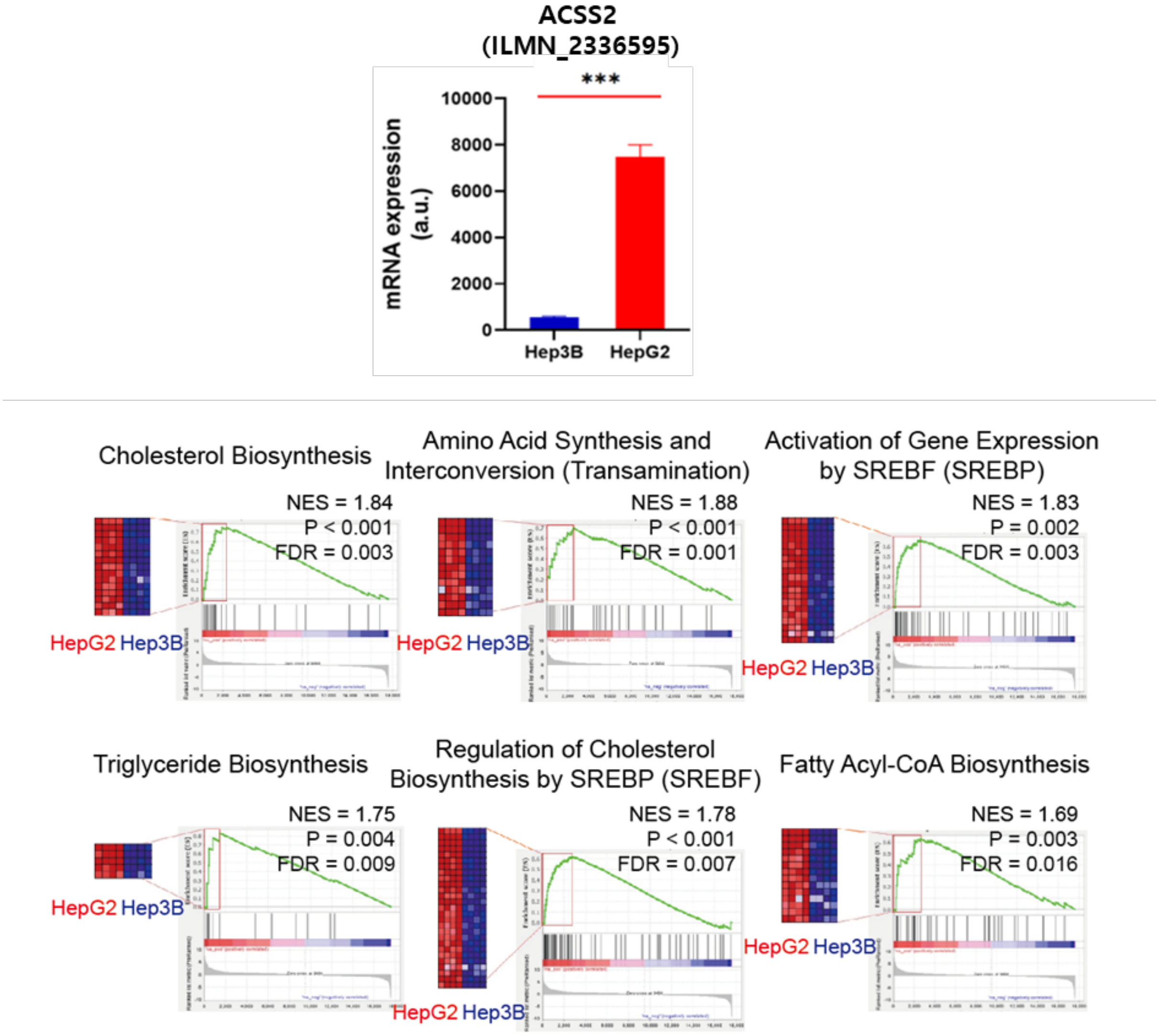

Supplementary Fig. 7

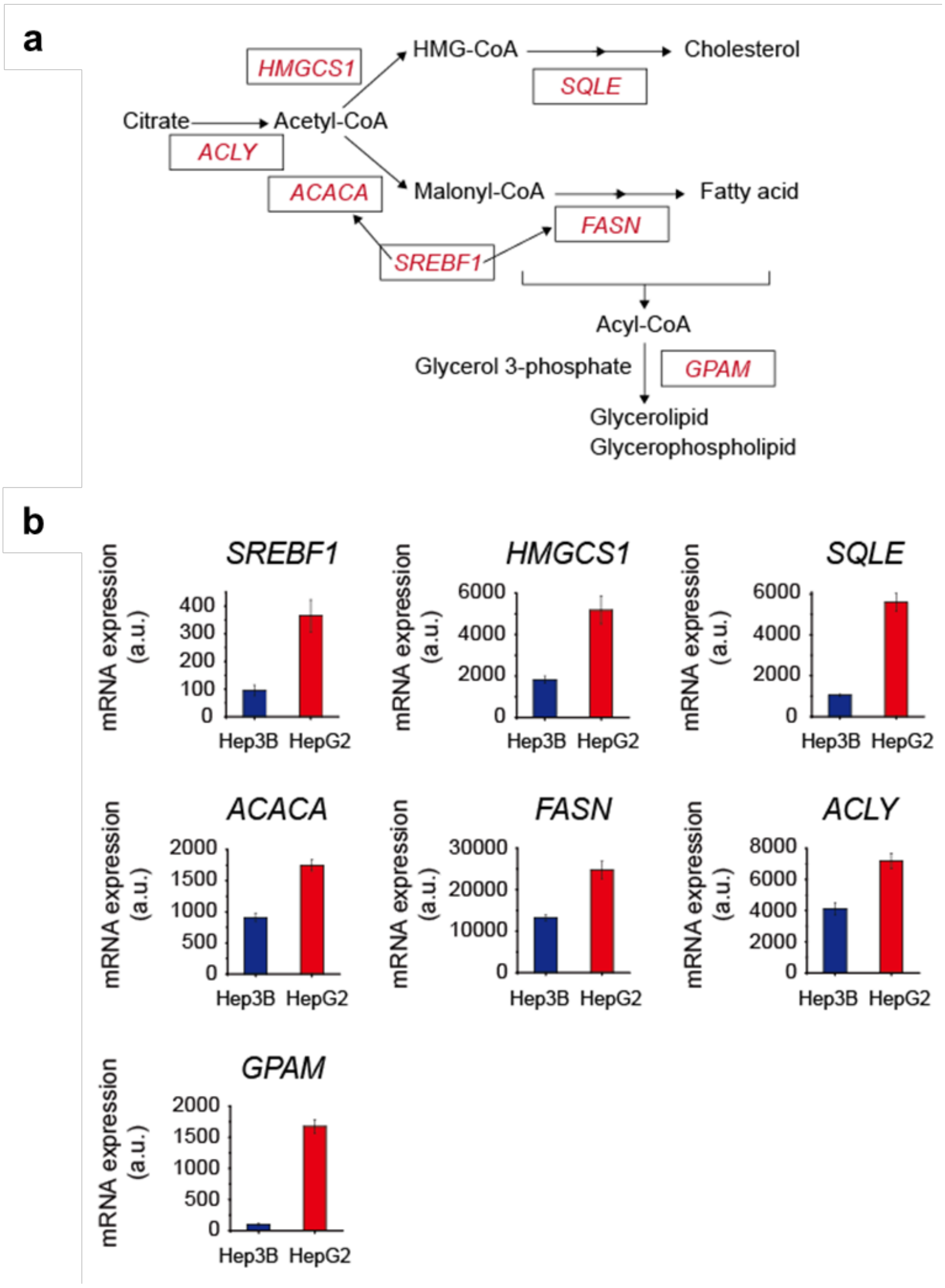

Supplementary Fig. 8

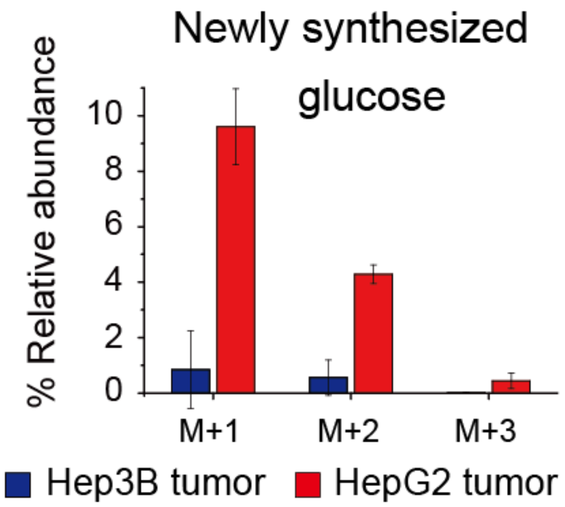

Supplementary Fig. 9

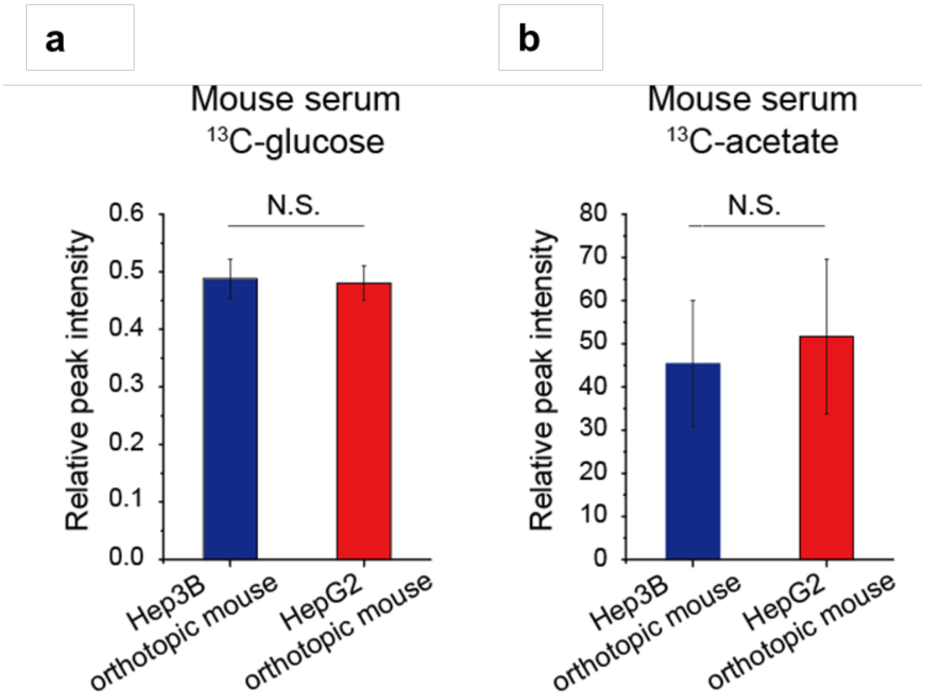

Supplementary Fig. 10

a

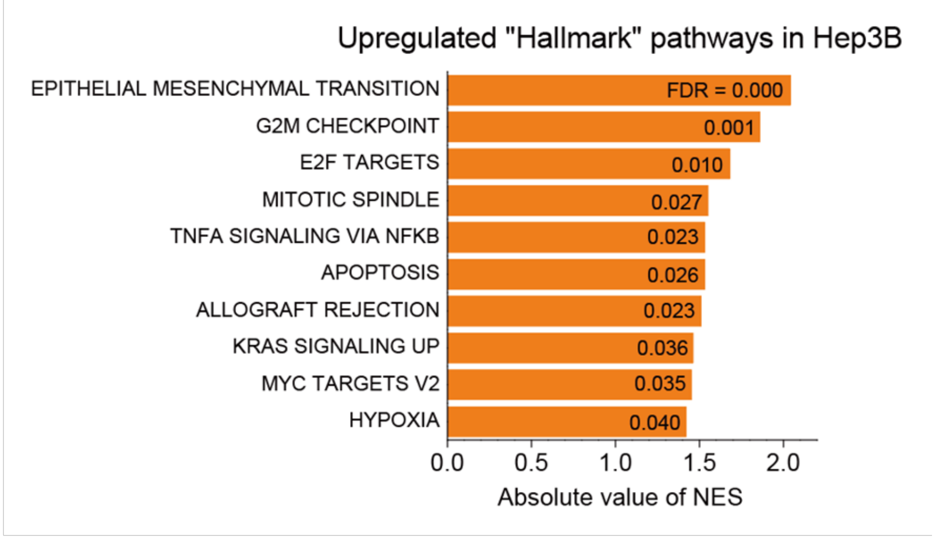

b

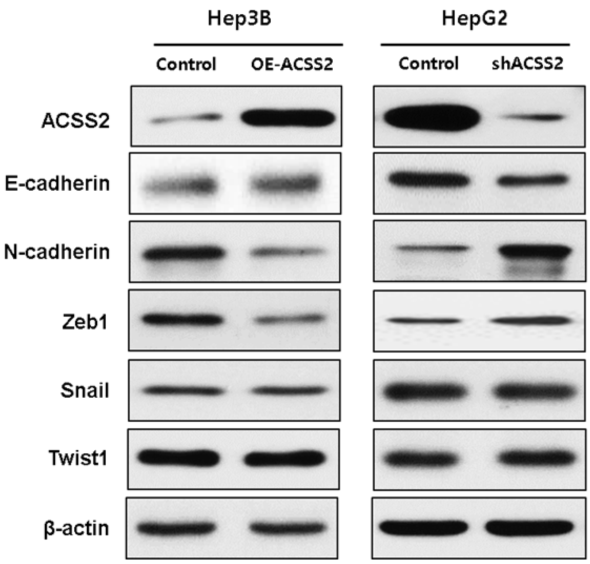

Supplementary Fig. 11

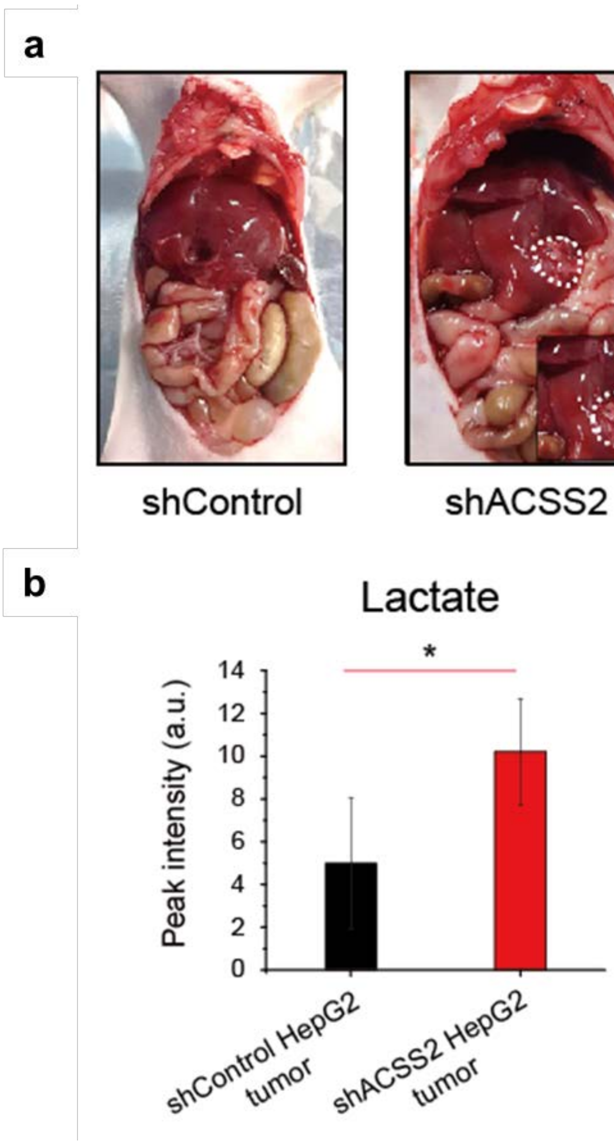

Supplementary Fig. 12

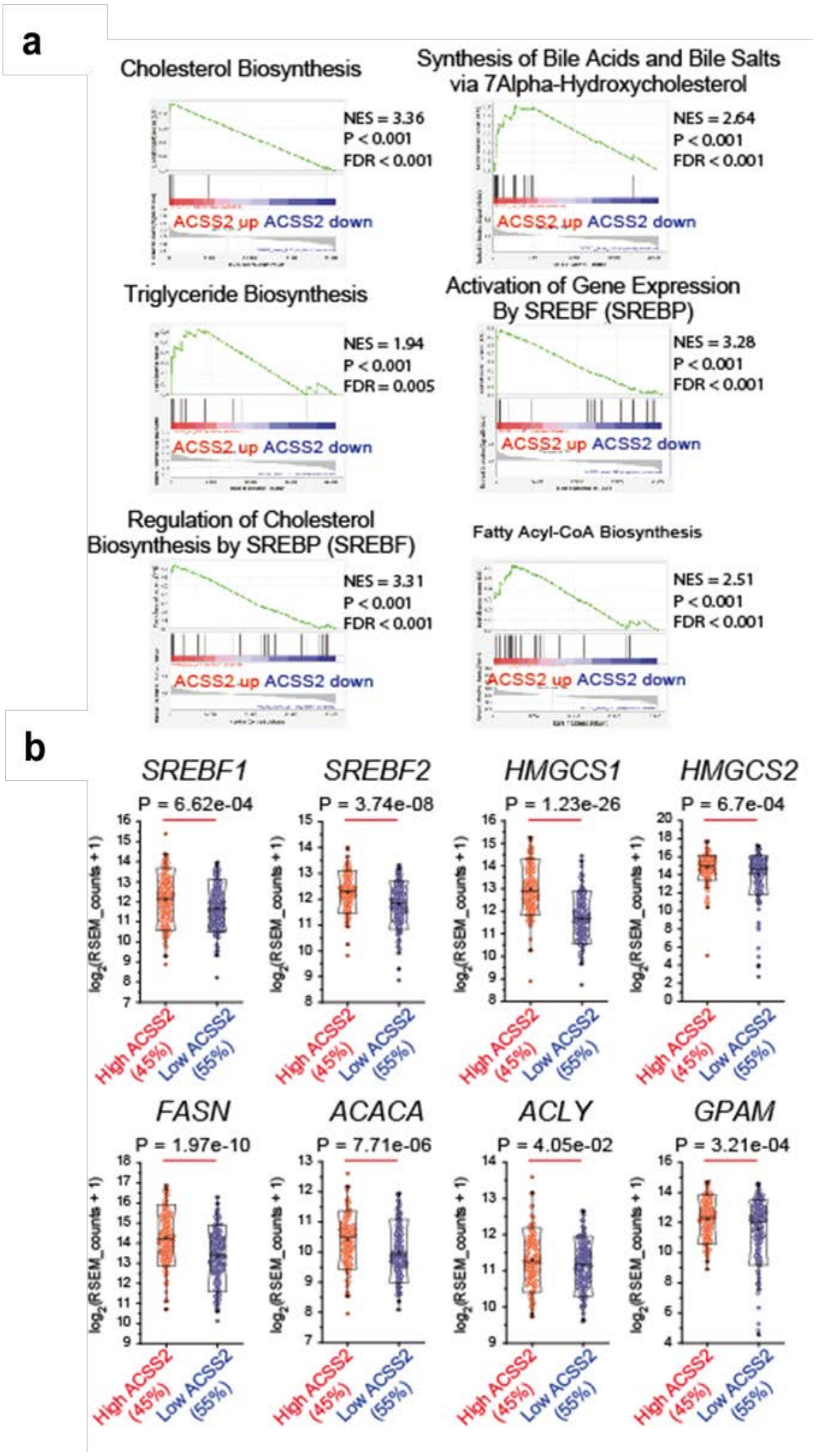

Supplementary Fig. 13

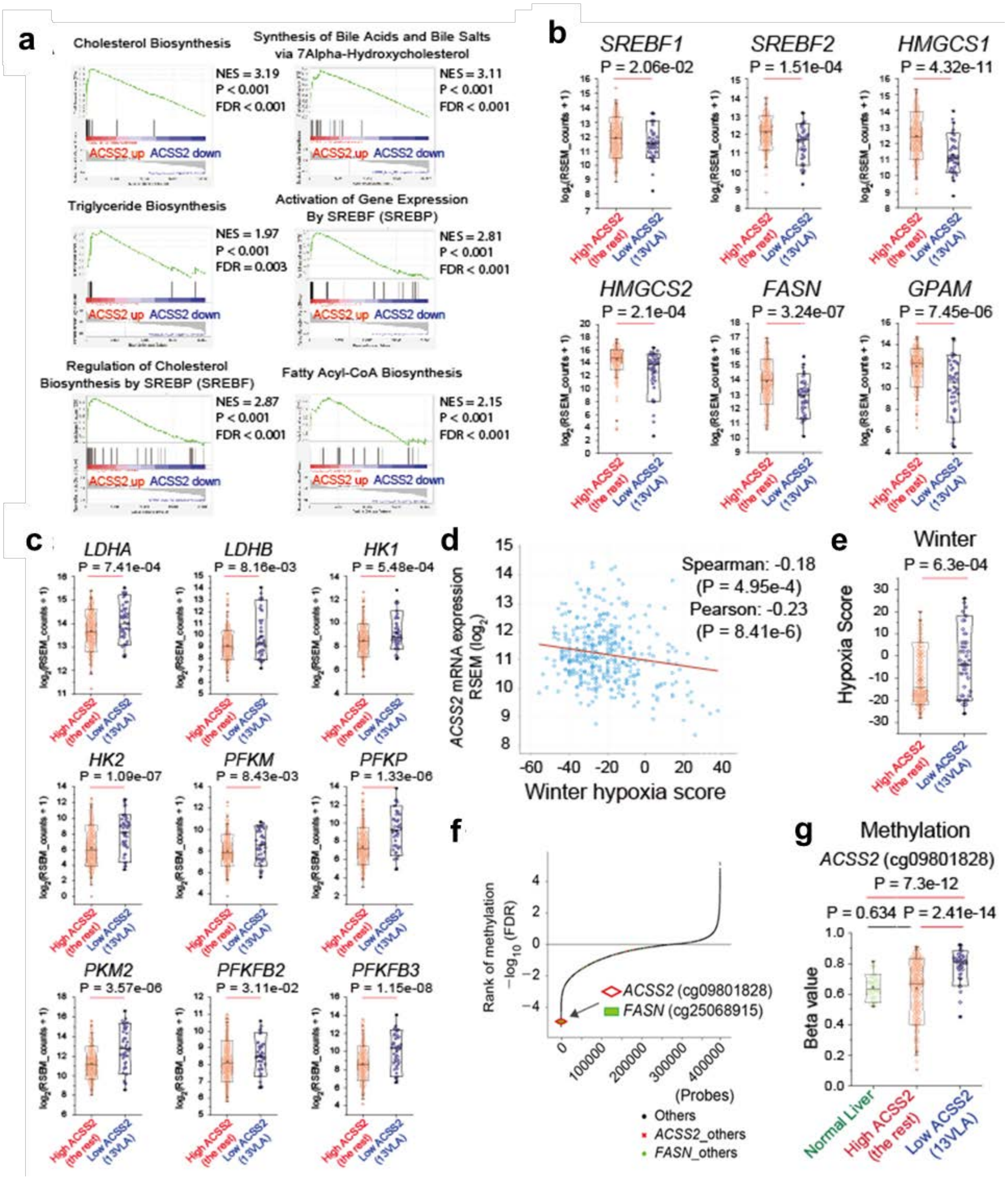

Supplementary Figure Legends

Supplementary Fig. 1 ACSS2 protein expression in liver tissues of DEN-induced HCC rats as tumor progresses. a Representative photo for whole liver and H&E staining of liver tissues of DEN-induced HCC rats. White dotted lines indicate the visible tumors which were excised and used for the staining and ACSS2 western blot. Expression of Ki-67 was measured in liver tumor tissue harvested at 0, 13, and 17 weeks. DEN-administered rats were sacrificed at indicated times. **b** Western blot for ACSS2 and β-actin control protein expression in liver tissues of normal and DEN-induced HCC rats. Normal tissues were obtained before the DEN-treatment.

Supplementary Fig. 2 Acetyl-CoA synthetase 1 (*ACSS1*) mRNA expression between 13VLA and the rest groups of the TCGA LIHC cohort.

Supplementary Fig. 3 PET-CT imaging of additional patients. A and B PET-CT imaging of low and high grade liver cancer patients.

Supplementary Fig. 4 Cell growth and migration in shACSS2 and OE-ACSS2 liver cancer cells. **a** Cell growth of shACSS2 and OE-ACSSE-liver cancer cells were determined by clonogenic assay after 14 days. **b** Wound-healing assay of OE-ACSSE Hep3B liver cancer cells. The cells were scratched to create a wound and migrated liver cancer cells were photographed at 200 x magnification.

Supplementary Fig. 5 2D HSQC NMR spectrum showing peak splitting by ^{13}C-^{13}C *J*-coupling.

Supplementary Fig. 6 GSEA results of gene expression related to lipid synthesis pathways in Hep3B and HepG2 cells in GSE21955 dataset. To validate the difference in lipid synthesis, we used independent transcriptomic dataset of HepG2 and Hep3B to determine the expression levels of ACSS2 (Top). Also, the control groups with the negative control siRNA for both cells were used for the analysis.

Supplementary Fig. 7 G**ene expression analysis of the lipid synthesis pathways in Hep3B and HepG2 cells in GSE21955 dataset**. **a** Major metabolites and genes for various lipid synthesis pathways. **b** Differential expressions of individual genes in (a) from the GSE21955 dataset. The error bars represent standard deviations.

Supplementary Fig. 8 Newly synthesized glucose from the Hep3B or HepG2 liver orthotopic cancer mice. Mice were orally administered with 3 g/kg 1,2-$^{13}C_2$-acetate, and the glucose isotopomers were measured with LC-MS.

Supplementary Fig. 9 Serum ^{13}C-glucose or ^{13}C-acetate levels of Hep3B or HepG2 liver orthotopic cancer mice. a and **b** ^{13}C-glucose or ^{13}C-acetate isotopic tracers were administered orally to the mice, and the levels of isotopically labeled tracers in the serum were measured by 800 MHz NMR. N.S.: not significant.

Supplementary Fig. 10 Top 10 "Hallmark" pathways upregulated in Hep3B cell line compared to HepG2 cell line in GSE21955 dataset. **a** The pathways were ordered by the absolute value of NES (Normalized Enrichment Score) in GSEA result. The "Hallmark" pathways were retrieved from MSigDB (Molecular Signatures Database). **b** EMT signaling in shACSS2 HepG2 and OE-ACSS2 Hep3B cells.

Supplementary Fig. 11 Tumorigenesis and lactate formation in orthotopic cancer mice with control or ACSS2 shRNA HepG2 cells. a Representative photo for liver tumor for each group. White dotted lines denote tumor. Inset is for the tumor region. **b** Lactate levels in the tumor tissues of control or ACSS2 shRNA HepG2 orthotopic mice. Lactate levels were measured with LC-MS.

Supplementary Fig. 12 Comparison of metabolic characters between the two groups according to 2352 RSEM cut-off value. a GSEA results for lipid-related pathways between the groups according to the 2352 RSEM cut-off value. NES, Normalized Enrichment Score; FDR, False Discovery Rate. **b** mRNA expressions of some key genes in the lipid anabolic pathways between the groups according to the 2352 RSEM cut-off value.

Supplementary Fig. 13 Metabolic characters of ACSS2-high and -low human liver cancer. a GSEA results for lipid-related pathways between the 13VLA group and the rest. NES, Normalized Enrichment Score; FDR, False Discovery Rate. **b** mRNA expressions of some key genes in the lipid anabolic pathways between the 13VLA group and the rest. **c** mRNA expressions for key genes in glycolysis between the 13VLA group and the rest. **d** Correlation between the *ACSS2* expression and the Winter hypoxia score for the entire TCGA LIHC cohort. **e** Winter hypoxia score between the 13VLA group and the rest. **f** The differential methylation rank plot

between the 13VLA group and the rest. ~390,000 methylation probes (sites) were ranked according to the FDR values. **g** The methylation beta values for the normal, 13VLA group, and the rest in the TCGA LIHC cohort.
