# Supplementary material for: Acetyl-CoA synthetase 2 contributes to a better prognosis for liver cancer by switching acetate-glucose metabolism
Source: Exp Mol Med. 2024 Mar 25;56(3):721–33. doi: 10.1038/s12276-024-01185-3 (PMC10984961; doi:10.1038/s12276-024-01185-3)
Supplement: Supplementary file 2 — Supplementary Table 1 [file 12276_2024_1185_MOESM2_ESM.docx]

**Supplementary Table 1. Primers sequence used for SYBR-green qRT-PCR**

| **Gene** | **Gene ID** | **Forward primer** | **Reverse primer** |
| --- | --- | --- | --- |
| ACSS2 | 55902 | GGTGACCAAGTTCTACACAGCAC | GTTCACCCACTGTGCCTAACAC |
| GAPDH | 2597 | GTCTCCTCTGACTTCAACAGCG | ACCACCCTGTTGCTGTAGCCAA |
